# Digital Interventions for Reducing Loneliness and Depression in Korean College Students: Mixed Methods Evaluation

**DOI:** 10.2196/58791

**Published:** 2024-09-12

**Authors:** Boyoung Kang, Munpyo Hong

**Affiliations:** 1 Sungkyunkwan University Seoul Republic of Korea

**Keywords:** loneliness, depression, digital interventions, college students, mental health, mixed methods evaluation, Woebot, Happify

## Abstract

**Background:**

The COVID-19 pandemic has exacerbated the prevalence of loneliness and depression among college students. Digital interventions, such as Woebot (Woebot Health, Inc) and Happify (Twill Inc), have shown promise in alleviating these symptoms.

**Objective:**

This study aims to investigate the effectiveness and acceptability of Woebot and Happify in reducing loneliness and depression among college students after the COVID-19 pandemic.

**Methods:**

A mixed methods approach was used over 4 months. A total of 63 participants aged 18 to 27 years from Sungkyunkwan University in Seoul, South Korea, were initially recruited, with an inclusion criterion of University of California, Los Angeles (UCLA) Loneliness Scale score ≥34. The final sample consisted of 27 participants due to attrition. Participants were randomly assigned to Woebot (15/27, 55%); Happify (9/27, 33%); or a control group using Bondee (Metadream), a metaverse social network messenger app (3/27, 11%). Quantitative measures (UCLA Loneliness Scale and Patient Health Questionnaire-9) and qualitative assessments (user feedback and focused interviews) were used.

**Results:**

Although mean decreases in loneliness and depression were observed in the control and intervention groups after the intervention, the differences between the control and intervention groups were not statistically significant (UCLA Loneliness: *P*=.67; Patient Health Questionnaire-9: *P*=.35). Qualitative data indicated user satisfaction, with suggestions for improved app effectiveness and personalization.

**Conclusions:**

Despite limitations, this study highlights the potential of well-designed digital interventions in alleviating college students’ loneliness and depression. The findings contribute to the growing body of research on accessible digital mental health tools and underscore the importance of comprehensive support systems. Further research with larger and more diverse samples is needed to better understand the effectiveness and optimization of such interventions.

**Trial Registration:**

Clinical Research Information Service KCT0009449; https://bit.ly/4d2e4Bu

## Introduction

### Background

The rising rates of depression and anxiety among college students worldwide, further compounded by the effects of the COVID-19 pandemic, emphasize the critical need for effective interventions to address these mental health challenges. In South Korea, the proportion of people in their 20s and 30s at the risk of depression increased 6-folds, to 22.8%, in 2021 compared to 2018 [1]. Similarly, in the United States, anxiety and depression symptoms among college students rose by 75% from 2019 to 2021 [2]. However, this trend is not solely attributable to the pandemic; college students’ mental health has been consistently declining over the past decade [3].

Depression, a potentially fatal illness, is a leading cause of suicide, claiming approximately 800,000 lives worldwide each year [4]. South Korea’s suicide rate surpasses the Organization for Economic Cooperation and Development average, with a notable increase in suicide rates among patients with depression [5,6]. The seriousness of depression is underscored by its high mortality rate; complex etiology involving genetic, environmental, and psychological factors; and the persistent stigma surrounding mental health [4-6].

Social isolation and loneliness are significant risk factors for mortality [7] and are particularly prevalent among college students [8-10]. The college years are a period of heightened emotional vulnerability, with students being particularly susceptible to depression and loneliness [11,12]. Loneliness can lead to social withdrawal and sleep disturbances and exacerbate depressive symptoms [8,13]. Hawkley and Cacioppo [13] demonstrated that loneliness can indeed lead to depression, particularly in young adults. Their research suggests that even when controlling for factors such as stress and social support, loneliness remains a predictor of depression in college students [13]. The correlation between loneliness and depression is partially attributed to changes in neurotransmitters that regulate mood, such as serotonin and dopamine [14]. In addition, loneliness can trigger negative coping behaviors such as alcohol and drug use, further exacerbating feelings of depression [8].

Cacioppo and Hawkley [15] and Matthews et al [9] emphasized the importance of addressing loneliness and depression in young adults for their overall well-being and development. Untreated depression in this age group can lead to academic difficulties, interpersonal relationship issues, and increased risk of self-harm or suicide. Timely intervention is essential to prevent progression to more severe or chronic depression. Various intervention methods suggest that loneliness and depression in young adults can be effectively alleviated. Key to these interventions is social support through meaningful connections formed through family, friends, school, or community activities [10]. The college years are crucial in career development stages and characterized by increased independence, intimacy, and the dynamic exploration of life goals [11,16]. However, college students often face challenges such as stress while adapting to new environments, anxiety about academic and career paths, and difficulties in relationships, all of which can negatively impact their psychological and emotional well-being [11,12]. Despite these challenges, the college years also provide unprecedented opportunities for intellectual growth and development [17,18].

Digital mental health interventions have shown promise in overcoming the limitations of traditional mental health services [19]. Grounded in theories such as self-determination theory and cognitive behavioral therapy (CBT), digital interventions have demonstrated effectiveness in reducing symptoms of depression and anxiety [19,20]. Self-determination theory emphasizes the importance of autonomy, competence, and relatedness in engaging individuals and initiating sustained changes [21], while CBT posits that depression arises from negative thoughts and beliefs, which can be modified through cognitive restructuring and behavioral activation [22]. The anonymity and accessibility of digital interventions make them particularly appealing to digital-native college students [23].

### This Study

This study explores the feasibility and acceptability of 2 digital therapy apps, Woebot (Woebot Health, Inc) and Happify (Twill Inc), among college students experiencing loneliness. Inspired by the research of Lim et al [24] on +Connect, this study has a larger sample size and an extended intervention period for a comprehensive evaluation. In addition, the research by Boucher et al [25] and Fitzpatrick et al [26] provided a foundation for selecting these specific digital interventions. The primary aim was to determine whether these digital interventions can meaningfully reduce feelings of loneliness and depression among college students in South Korea. The study also investigated the role of participants’ beliefs and behaviors in the effectiveness of the interventions. The hypotheses were as follows:

- Hypothesis 1: Participants using Woebot and Happify will show a greater reduction in loneliness and depression scores compared to the control group using the Bondee (Metadream) app.
- Hypothesis 2: Participants who expect this intervention experiment to reduce loneliness and depression will experience greater reductions in these symptoms (perceived benefits of action).
- Hypothesis 3: Participants who believe that even digital applications can alleviate loneliness and depression will experience greater reductions in these symptoms (perceived barriers to action).
- Hypothesis 4: Participants with low help-seeking behavior will experience greater reductions in loneliness and depression after using the digital interventions (help-seeking behavior).

In addition, qualitative feedback will provide insights into the user experience, perceived effectiveness, and areas for improvement of these digital interventions.

## Methods

### Experimental Design

This study aimed to explore the feasibility and acceptability of digital interventions in reducing loneliness and depression among university students. The study was conducted from March to June 2023, over a 4-month period, with participants from Sungkyunkwan University’s Colleges of Natural Sciences and Humanities and Social Sciences in Seoul, South Korea. The study used a mixed methods approach, incorporating both quantitative and qualitative data collection and analyses. The clinical trial was registered with the Clinical Research Information Service, a World Health Organization International Clinical Trials Registry Platform–linked registry, under the registration KCT0009449.

### Intervention

Given the importance of selecting effective digital interventions for addressing loneliness and depression among college students, Woebot and Happify were chosen for their demonstrated efficacy in prior research. Participants were provided with an instruction manual (Multimedia Appendix 1) to guide them through the use of the assigned intervention app. Woebot, a chatbot based on CBT principles, has shown effectiveness in reducing symptoms of depression and anxiety among young adults, as evidenced by studies such as the randomized controlled trial conducted by Fitzpatrick et al [26]. In addition, Happify has demonstrated promise in alleviating loneliness, particularly during challenging periods such as the COVID-19 pandemic, as reported by Boucher et al [25].

Moreover, both Woebot and Happify are readily downloadable in South Korea, providing convenient and accessible platforms for individuals seeking to improve their mental well-being. These chatbots were also selected based on the performance results of other mental health chatbots that have been verified for effectiveness and satisfaction in clinical experimental studies. Building on the promising findings of a recent study by Li et al [27], regarding their top-tier performance in terms of engagement (Woebot: mean 4.10, SD 0.14; Happify: mean 4.60, SD 0.00), functionality (Woebot: mean 4.65, SD 0.21; Happify: mean 4.25, SD 0.00), and overall quality (Woebot: mean 4.31, SD 0.09; Happify: mean 4.15, SD 0.02) on a 5-point scale, Woebot and Happify were selected as the interventions for this study. The features and instructions of use of Woebot and Happify are shown in Table 1.

Given the lack of Korean-language versions of Woebot and Happify, all interactions with the apps were conducted in English. This decision was made to ensure uniformity across all intervention groups, despite the potential challenge it posed to participants with lower English proficiency. No special cultural adaptations for Korean users were implemented, although compensation was provided in the form of a choice between equivalent-value coffee coupons and cash, with all participants opting for the cash incentive.

**Table 1:** Digital interventions: Woebot and Happify.

	Woebot	Happify
Type of therapy	CBT^a^	CBT and mindfulness
Delivery method	Chatbot	Chatbot, games, and activities
Cost	Free	Free and premium (paid)
Availability	Use in academic research	Commercial use
Other features	Weekly mood description	Mood tracking, social community, and rewards
Benefits	Can help improve mood, reduce stress, and improve cognitive function	Can help improve mood, reduce stress, and improve cognitive function
Previous representative research	Fitzpatrick et al [26]	Boucher et al [25]
Instructions	Step 1: download the app from the app store or Google Play. Step 2: create an account by entering your email address, password, date of birth, and nickname. Step 3: open the app and use it for at least 15 minutes every day. You can choose from a variety of topics, including Focusing on Positive, Relationship, Mindfulness & Meditation, and Managing Emotions. You must also use the gratitude journal. Step 4: capture and save the journal 3 times a week. This is necessary for record keeping and monthly participant rewards.	Step 1: download the app from the app store or Google Play. Step 2: create an account by entering your sex, age, race, occupation, family relationships, and current mental state. Step 3: open the app and use it for at least 15 minutes every day. You can choose from a variety of activities in the Instant Play section, including Serenity Scene, Guided Meditation, and Negative Knockout. Thank mode is required for each activity. You can also use the Mindfulness & Meditation track (free). Step 4: capture and save the activity tasks 3 times a week. This is necessary for record keeping and monthly participant rewards.

^a^CBT: cognitive behavioral therapy.

### Participant Recruitment and Selection

The target sample size was based on previous studies on digital mental health interventions. Initially, 63 undergraduate and graduate students aged 18 to 27 years were recruited through the university portal (see Multimedia Appendix 2 for the recruitment notice and survey questionnaires). Participants with very low loneliness scores (<34) were excluded. While the recruitment notice mentioned that participants should be confident in using English, as the Woebot and Happify apps do not have Korean versions, no applicants were excluded based on their English proficiency. To ensure fairness, all participants, including those in the control group using Bondee, were required to interact with the apps in English. Due to high attrition rates, the final sample consisted of 27 participants (Woebot: n=15, 55%; Happify: n=9, 33%; Bondee: n=3, 11%). To enhance retention, participants received a US $30 incentive for completing the study and an additional US $30 for completing the postintervention assessment [28].

### Randomization

Participants were assigned to 1 of the 3 groups (Woebot, Happify, or Bondee) using a stratified randomization method. The participants’ names were blinded, and they grouped such that there were similar distributions of age, sex, and loneliness scores across the groups. The decision to have fewer participants in the control group was made to ensure sufficient power in the intervention groups, given the limited overall sample size and anticipated high attrition rates in the control group. This decision was made based on the intention of the research to ensure the study focused on the effectiveness of the interventions.

### Assessment Tools and Data Collection

#### Quantitative Data

Quantitative data were collected using the University of California, Los Angeles (UCLA) Loneliness Scale (Korean version) and the Patient Health Questionnaire-9 (PHQ-9; Korean-translated version) before and after the intervention (see Multimedia Appendix 3 for the questionnaires). The primary outcome was reduction in loneliness and depression scores. The secondary outcomes were user satisfaction and perceived benefits of the digital interventions.

#### Cronbach α

In addition to examining the quantitative outcomes, Cronbach α coefficients were calculated to assess the reliability of the measurement scales used in the study (Table 2). The consistently high Cronbach α coefficients (range 0.848-0.928) for the UCLA Loneliness Scale across all time points indicate strong internal consistency. This high internal consistency enhances the internal validity of the test and underscores the reliability of the loneliness scores obtained throughout the study. The robustness of the measurement instrument strengthens the confidence in the interpretation of loneliness levels among participants, thereby bolstering the internal validity of our experiment.

The PHQ-9 coefficients were generally lower due to the shorter scale length (9 items). However, all PHQ-9 coefficients, except for the baseline coefficient (0.680), were above the acceptable threshold of 0.6 for short scales [29].

**Table 2:** Internal consistency reliability of measurement scales: Cronbach α coefficients.

Measurement tool and time point		Cronbach α
**UCLA^a^ Loneliness Scale**		
	Baseline	0.848
	1-month follow-up	0.893
	2-month follow-up	0.928
	After the intervention	0.904
**PHQ-9^b^**		
	Baseline	0.680^c^
	1-month follow-up	0.702
	2-month follow-up	0.746
	After the intervention	0.769

^a^UCLA: University of California, Los Angeles.

^b^PHQ-9: Patient Health Questionnaire-9.

^c^The PHQ-9 coefficients were generally lower due to the shorter scale length (9 items). However, all PHQ-9 coefficients except for the baseline (0.680) were above the acceptable threshold of 0.6 for short scales [29].

#### Qualitative Data

Qualitative data were gathered through open-ended survey questions and 5 focus group interviews conducted via Zoom (Zoom Video Communications, Inc), each lasting approximately 90 minutes with 5 to 6 participants per group. Participants were given a preliminary orientation via KakaoTalk (Kakao Corp), a popular Korean social networking service, which included an introduction to the concept of focus group interviews, the methodology, and the anticipated questions. The interviews explored participants’ experiences and coping mechanisms for loneliness and app features. Participants were provided with an orientation document introducing the concept and methodology of focus group interviews as well as containing a list of anticipated questions. However, due to time constraints, the actual interviews primarily focused on a subset of these questions, that is, those related to coping with loneliness and user experiences with the apps (Multimedia Appendix 4).

### Interview Questions

The following questions were asked during the focus group interviews.

- What features of the app did you find most helpful?
- What challenges did you encounter while using the app?
- How did the app impact your feelings of loneliness and depression?
- What improvements would you suggest for the app?

### Data Analysis

Textbox 1 provides the list of statistics considered and tests conducted during qualitative and quantitative analyses.

**Summary of data analysis methods.**

**Quantitative analysis**

- Descriptive statistics: mean, median, IQR, and SD [30]
- Group comparisons: 2-tailed *t* tests for normally distributed data and Mann-Whitney *U* tests for nonnormally distributed data [31]
- Statistical software: data analysis was conducted using Python (*numpy*, *scipy*, and *matplotlib*) after extracting results from Google Surveys in CSV format ChatGPT was used to refine the data statistical analysis code.
- Significance level: α=.05
- CI: 95%
- The data analysis code used in this study is available in Multimedia Appendix 5.

**Qualitative analysis**

- Coding approach: an inductive approach was used to identify themes from the data [32]
- Software: Zoom was used for real-time recording, Naver Clova was used for transcription, and Python-based text mining was used to extract key themes and related content
- Process: themes were identified and refined through an iterative review of transcripts. The primary author conducted the coding, with periodic reviews by another researcher to ensure consistency AI tools, including Claude AI, were used to check for duplicates and assist in the refinement of the coding process.
- Validity and reliability: data validity and reliability were ensured through triangulation, member checking, and maintaining a detailed audit trail [29]
- Confidentiality: data were deidentified to maintain participant confidentiality
- Homogeneity: focus groups consisted of participants using the same app, primarily third-year university students
- Data saturation: achieved through repetitive themes identified across the focus groups

### Ethical Considerations

This study was approved by the institutional review board (IRB) of Sungkyunkwan University, Seoul, South Korea (IRB file: SKKU 2023-02-043; approval date: February 27, 2023; see Multimedia Appendix 6 for the IRB approval letter). All participants provided informed consent before their involvement in the study (see Multimedia Appendix 7 for the informed consent form). Data were deidentified to ensure confidentiality. Participants were compensated for their time and participation in the study. Compensation varied based on participation, with a maximum amount of up to KRW 200,000 (US $154). Participants received KRW 10,000 (US $7.70) per survey, KRW 20,000 (US $15.40) per interview, up to KRW 30,000 (US $23.10) for experimental participation, and an additional reward at the end of the study (amount to be determined). Compensation was calculated based on participation frequency and compliance, and payments were made at the end of each month after the interviews were completed. The details of this compensation were clearly outlined in the informed consent form provided to all participants.

### Consolidated Standards of Reporting Trials Reporting

**Figure 1:**
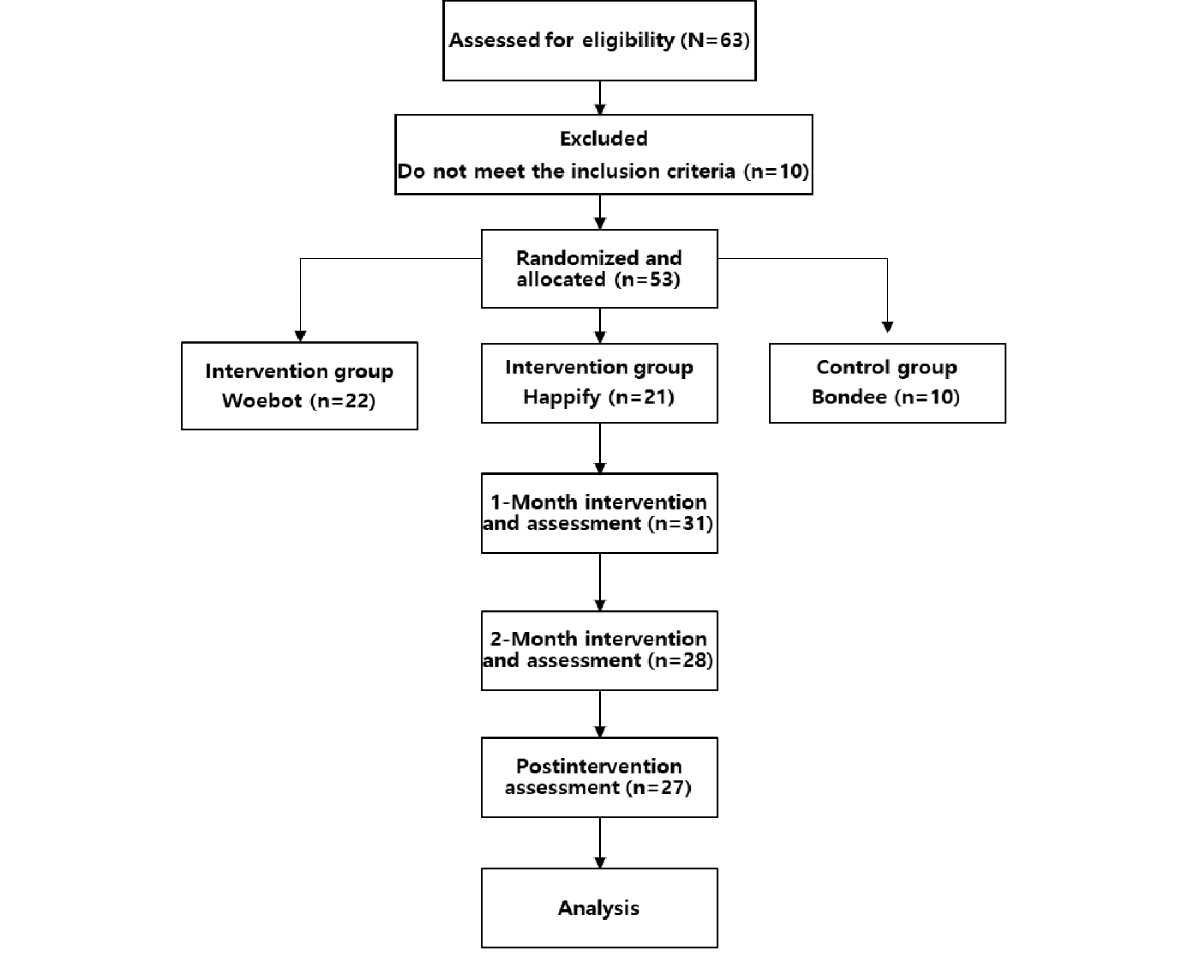
CONSORT (Consolidated Standards of Reporting Trials) flow diagram of participants.

This study followed the CONSORT (Consolidated Standards of Reporting Trials) reporting guidelines for randomized controlled trials. The CONSORT flow diagram, illustrating the flow of participants through the study, is presented in Figure 1. The CONSORT-EHEALTH (Consolidated Standards of Reporting Trials of Electronic and Mobile Health Applications and Online Telehealth) checklist (version 1.6) [33] was also completed and is available as Multimedia Appendix 8.

## Results

### Demographic and Clinical Variables of Participants at Baseline

Table 3 presents the demographic and clinical variables of participants at baseline. The table includes measures of central tendency and dispersion, such as mean, SD, median, and IQR for both the UCLA Loneliness Scale and PHQ-9.

Among the initial 63 applicants, the average PHQ-9 score was 9.23 (SD 6.15), with 25 (40%) individuals classified as being at risk for depression (PHQ-9≥10). At the baseline survey, 23 (43%) out of 53 participants were categorized as being at risk for depression, which is considered a very high proportion according to the Ministry of Health and Welfare’s criteria [1]. These findings highlight the urgent need for interventions to address the mental health crisis among college students.

In addition, participants with a UCLA Loneliness Scale score of ≥65, indicating severe loneliness, and those with a PHQ-9 score of ≥20, indicating severe depression, were advised to seek further counseling.

**Table 3:** Demographic and clinical variables of participants at baseline (n=53).

Variable		Woebot (n=22)	Happify (n=21)	Control (Bondee) (n=10)
**UCLA^a^ Loneliness**				
	Values, mean (SD)	53.18 (9.14)	53.67 (11.05)	53.50 (7.09)
	Values, median (IQR)	53.5 (47-59)	53.0 (45-62)	50.5 (48-59)
**Depression (PHQ-9^b^)**				
	Values, mean (SD)	10.55 (5.89)	9.14 (5.84)	12.50 (7.47)
	Values, median (IQR)	9.5 (7-12)	8.0 (4-11)	12.5 (8-15)
**Age (y)**				
	Values, mean (SD)	23.5 (1.78)	23.0 (2.14)	22.9 (1.85)
	Values, median (IQR)	22 (21-25)	23 (22-24)	23 (22-24)
**Sex, n (%)**				
	Female	14 (64)	11 (52)	5 (50)
	Male	7 (32)	9 (43)	5 (50)
	Prefer not to disclose	1 (5)	1 (5)	0 (0)
**Majors, n (%)**				
	Humanities and social science	11 (50)	10 (48)	4 (40)
	Natural science and engineering	11 (50)	11 (52)	6 (60)

^a^UCLA: University of California, Los Angeles.

^b^PHQ-9: Patient health Questionnaire-9.

### Participant Flow

Figure 1 displays the CONSORT flow diagram, illustrating the flow of participants through the study. Of the initial 63 respondents, 10 (16%) were excluded due to loneliness scores <34. The remaining 53 (84%) participants were randomly assigned to the intervention groups (Woebot: n=22, 42%; Happify: n=21, 40%) and the control group (Bondee: n=10, 19%). Due to attrition, the final sample consisted of 27 participants (Woebot: n=15, 55%; Happify: n=9, 33%; Bondee: n=3, 11%).

### Quantitative Findings

#### Intervention Effects and Trends

**Figure 2:**
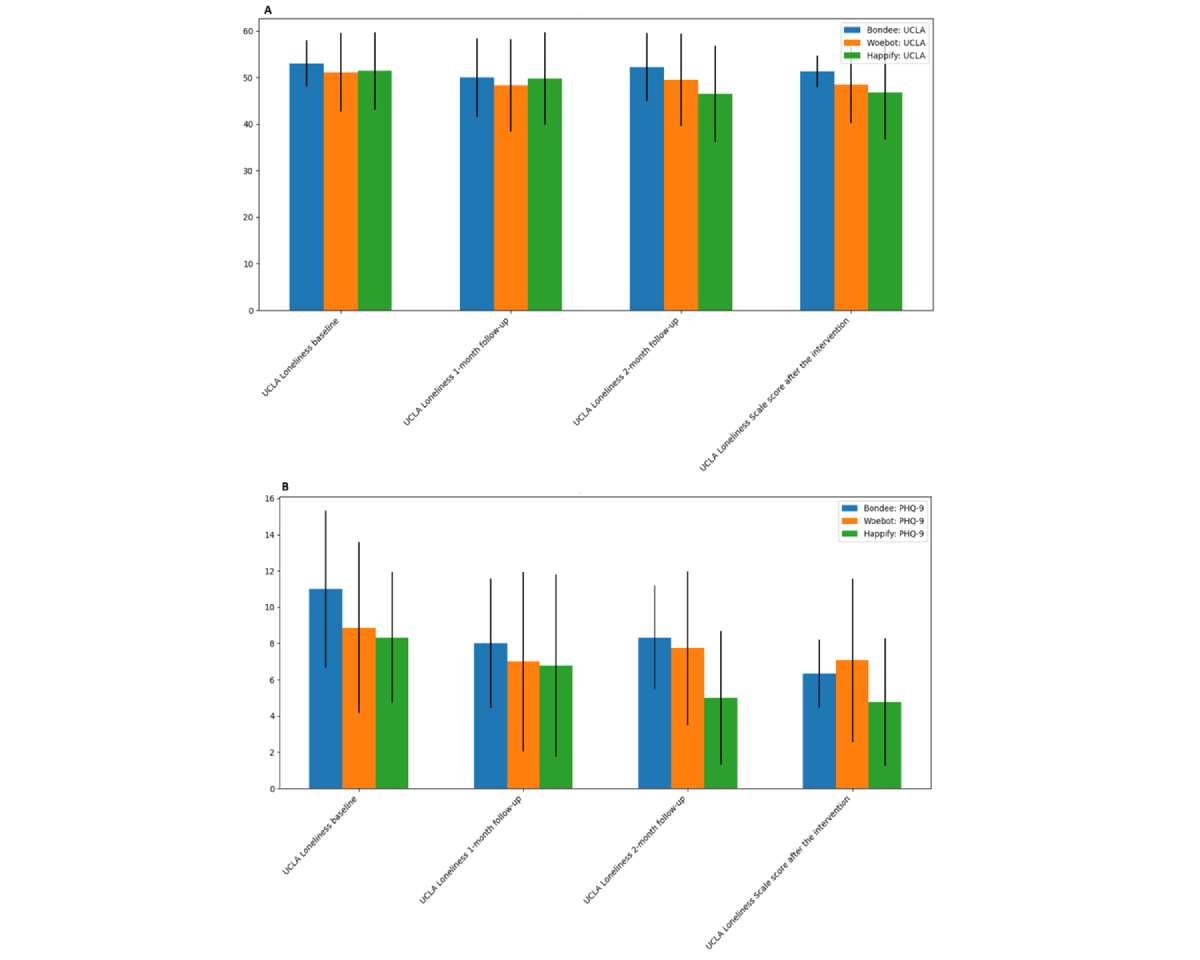
(A) University of California, Los Angeles (UCLA) Loneliness Scale and (B) Patient Health Questionnaire-9 (PHQ-9) trend charts.

The quantitative data analysis results, presented in Figure 2 and Table 4, show decreases in loneliness and depression after the intervention. However, these effects did not reach statistical significance, likely due to the small sample size. The control group (Bondee) saw a significant decrease in user numbers (baseline: n=10; after the intervention: n=3), complicating comparisons. Consequently, hypothesis 1 was not verified.

To address the unbalanced sample sizes (Bondee: 3/27, 11%; Woebot: 15/27, 55%; Happify: 9/27, 33%), Welch ANOVA was used, as it is robust to violations of equal variances. However, the overall small sample size limited the power to detect significant effects, and the quantitative results should be interpreted with caution.

**Table 4:** Descriptive statistics and group comparisons for University of California, Los Angeles (UCLA) Loneliness Scale and Patient Health Questionnaire-9 (PHQ-9) scores^a^.

Variable and time point		Woebot (n=15), mean (SD)	Happify (n=9), mean (SD)	Control (Bondee; n=3), mean (SD)	Group comparison test	*P* value
**UCLA Loneliness**						
	Baseline	51.13 (8.73)	51.44 (8.76)	53 (6.08)	*t* test	.93
	1 month	48.40 (10.27)	49.78 (10.51)	50 (10.39)	Mann-Whitney *U*	.68
	2 months	49.53 (10.26)	46.56 (10.72)	52.33 (8.96)	*t* test	.51
	Postintervention	48.47 (10.26)	46.78 (10.92)	51.33 (4.16)	*t* test	.67
**PHQ-9 Depression**						
	Baseline	8.87 (4.88)	8.33 (3.81)	11 (5.29)	*t* test	.78
	1 month	7 (5.13)	6.78 (5.33)	8 (4.36)	Mann-Whitney *U*	.83
	2 months	7.73 (4.38)	5 (3.91)	8.33 (3.51)	*t* test	.14
	Postintervention	7.07 (4.67)	4.78 (3.73)	6.33 (3.21)	Mann-Whitney *U*	.35

^a^*P* values indicate the significance level of group comparisons. α level was set at.05. *t* test (2-tailed) was used for normally distributed data with equal variances. Mann-Whitney *U* test was used when data did not meet normal distribution criteria. Detailed data analysis procedures are provided in the Patient Health Questionnaire-9 (PHQ-9) and University of California, Los Angeles (UCLA) Loneliness Trend Analysis code in Multimedia Appendix 5.

#### Description of Results

##### UCLA Loneliness Scale

In control group using Bondee, the mean UCLA Loneliness Scale score remained relatively stable, from 53 (SD 6.08) at baseline to 51.33 (SD 4.16) after the intervention. The group comparison test (*t* test) did not show a statistically significant difference among the 3 groups at baseline (*P*=.93) or after the intervention (*P*=.67). In the group using Woebot, the mean UCLA Loneliness Scale score decreased from 51.13 (SD 8.73) at baseline to 48.47 (SD 10.26) after the intervention. In the group using Happify, the mean UCLA Loneliness Scale score decreased from 51.44 (SD 8.76) at baseline to 46.78 (SD 10.92) after the intervention.

##### PHQ-9 Depression Scale

In the control group using Bondee, the mean PHQ-9 depression score decreased from 11 (SD 5.29) at baseline to 6.33 (SD 3.21) after the intervention. The group comparison test (*t* test) did not show a statistically significant difference among the 3 groups at baseline (*P*=.78), and the Mann-Whitney *U* test did not show a significant difference between the control and intervention groups after the intervention (*P*=.35). In the group using Woebot, the mean PHQ-9 depression score decreased from 8.87 (SD 4.88) at baseline to 7.07 (SD 4.67) after the intervention. In the group using Happify, the mean PHQ-9 depression score decreased from 8.33 (SD 3.81) at baseline to 4.78 (SD 3.73) after the intervention.

Overall, although the intervention groups (using Woebot and Happify) showed decreases in both UCLA Loneliness Scale and PHQ-9 depression scores from baseline to after the intervention, the group comparison tests did not reveal statistically significant differences among the groups either at baseline or after the intervention for both measures.

#### Hypothesis Testing

As hypothesized in the *Introduction* section, we investigated the effectiveness of the digital interventions and the role of participants’ beliefs and behaviors in their outcomes.

##### Hypothesis 1: Intervention Effect

The quantitative results showed decreases in both UCLA Loneliness Scale and PHQ-9 depression scores from baseline to after the intervention in the intervention groups (using Woebot and Happify). However, the group comparison tests (*t* test for normally distributed data and Mann-Whitney *U* test for nonnormally distributed data) did not reveal statistically significant differences between the control and intervention groups after the intervention (UCLA Loneliness Scale: *P*=.67; PHQ-9 depression: *P*=.35). Therefore, hypothesis 1 was not supported.

##### Hypothesis 2: Perceived Benefits of Action

The positive group, which expected this intervention experiment to reduce loneliness and depression, showed higher reduction rates in scores than the negative group. However, Mann-Whitney *U* tests did not reveal statistically significant differences between the groups (UCLA Loneliness Scale: *P*=.93; PHQ-9: *P*=.48). Therefore, hypothesis 2 was not supported.

##### Hypothesis 3: Perceived Barriers to Action

For participants believing in the effectiveness of digital applications in general, the difference in UCLA Loneliness Scale scores (*P*=.60) and PHQ-9 scores (*P*=.47) between the positive and negative groups was not statistically significant according to Mann-Whitney *U* tests. Therefore, hypothesis 3 was not supported.

##### Hypothesis 4: Help-Seeking Behavior

The results for self-help behavior attitudes showed nonsignificant differences between the groups (UCLA Loneliness Scale: *P*=.34; PHQ-9: *P*=.62) based on Mann-Whitney *U* tests. Therefore, hypothesis 4 was not supported.

It is important to note that the small and unbalanced sample sizes across the study groups may have limited the statistical power and generalizability of the hypothesis tests. The notably smaller control group compared to the intervention groups, as well as the overall limited sample size, could have influenced the nonsignificant results. These limitations should be taken into account when interpreting the findings, and future studies with larger and more balanced samples are needed to further investigate these hypotheses and to obtain more conclusive results.

**Figure 3:**
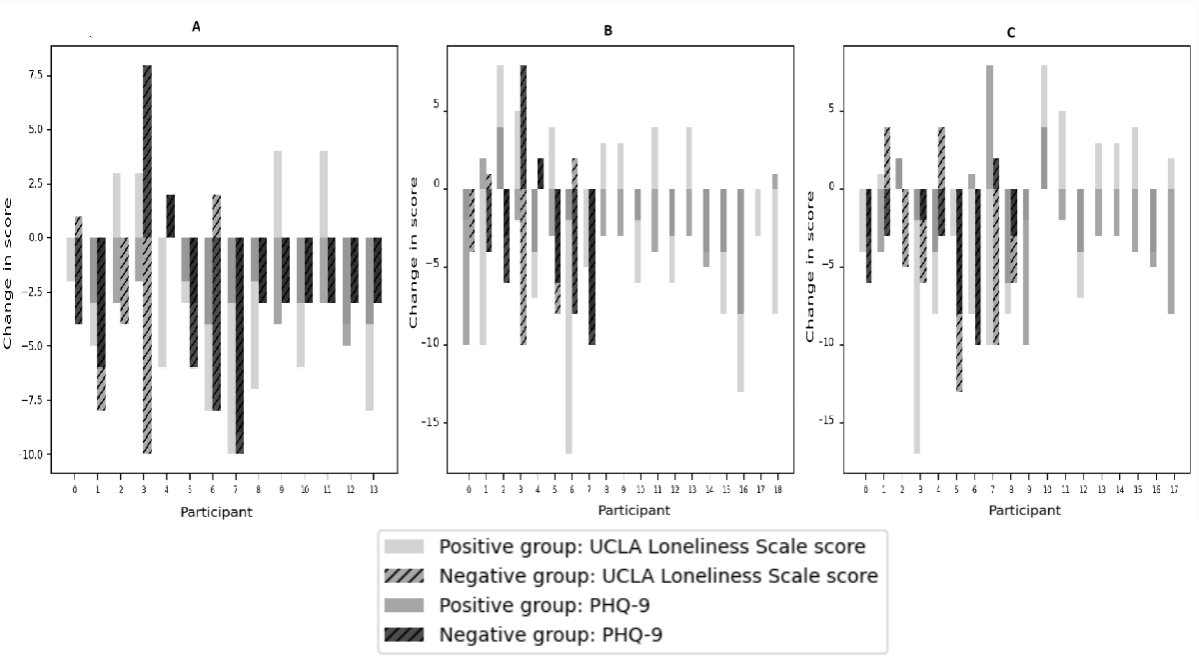
Health behavior belief versus loneliness and depression. (A) Hypothesis 2: perceived benefits of action (the positive group is the group of people who answered, “I expected my loneliness and depression would decrease after the experiment”). (B) Hypothesis 3: perceived barriers to action (the positive group is the group of people who answered, “even digital application can help people relieve loneliness”). (C) Hypothesis 4: help-seeking behavior (the positive group is the group of people who answered, “it is difficult to control loneliness and depression on one’s own will and efforts”). PHQ-9: Patient Health Questionnaire-9; UCLA: University of California, Los Angeles.

Figure 3 visually presents the comparisons between the positive and negative groups for each of the hypotheses (hypotheses 2, 3, and 4). For hypotheses 2 (perceived benefits of action), 3 (perceived barriers to action), and 4 (help-seeking behavior), the differences between the positive and negative groups were not statistically significant for either UCLA Loneliness Scale or PHQ-9 depression scores, as indicated by the *P* values >.05.

In summary, although the intervention groups showed reductions in loneliness and depression scores, the quantitative analyses did not provide statistically significant evidence to support the hypothesized intervention effect (hypothesis 1) or the influence of participants’ beliefs and behaviors on the outcomes (hypotheses 2, 3, and 4). The nonsignificant results should be interpreted with caution due to the limitations imposed by the small sample size.

From the quantitative analysis, we added the self-reported mental health literacy levels and app satisfaction ratings. The results are described in the subsequent sections.

#### Self-Reported Cohort Mental Health Literacy

**Figure 4:**
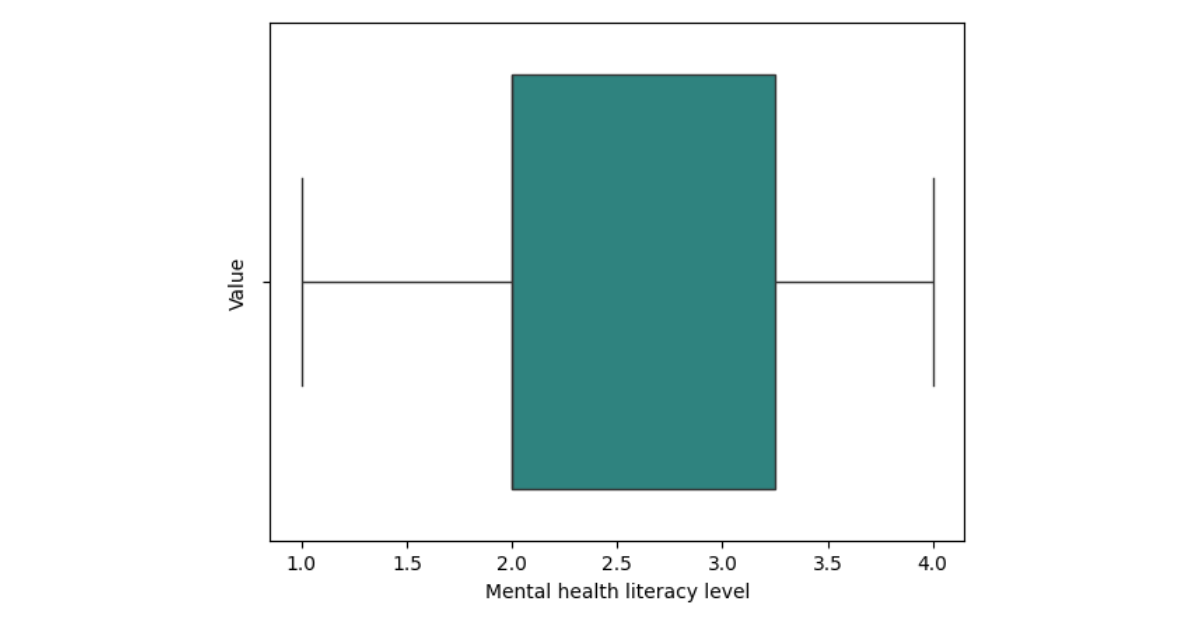
Self-reported cohort mental health literacy.

Participants rated their mental health literacy on a 5-point scale (1=poor, 5=abundant). The mean score was 2.46, indicating relatively low self-perceived mental health literacy (Figure 4).

#### App Satisfaction Rating

User satisfaction was moderate (Table 5). Most users (16/24, 67%) were somewhat satisfied, with few (2/24, 8%) reporting high satisfaction. Low retention rates suggest that even somewhat satisfied users may not have found the apps compelling enough for continued use.

**Table 5:** Satisfaction ratingsa.

Question	Very satisfied			Somewhat satisfied			Not satisfied	
	Woebot	Happify	Woebot		Happify	Woebot		Happify
Overall satisfaction	1	0	12		4	2		6
Easy to use	0	0	11		4	4		8
Novelty	0	0	5		2	10		7
Effectiveness	2	0	11		5	2		4
Intention to maintain	0	0	6		3	9		6

^a^Not satisfied=1-3, somewhat satisfied=4-7, and very satisfied=8-10.

#### Interpretation of Nonsignificant Results

Despite nonsignificant quantitative results, qualitative insights highlight practical benefits and potential improvements for the interventions.

### Qualitative Findings

The qualitative findings from the open-ended survey questions and focus group interviews provided valuable insights into participants’ experiences with the digital interventions and their coping mechanisms for loneliness.

#### Favored App Features and Areas for Improvement

Participants highlighted favored features and areas for improvement in the digital interventions. Woebot users appreciated the app’s responsiveness and tone, while Happify users valued features supporting meditation and gratitude practice (Table 6). Suggestions for improvement included enhancing app effectiveness, personalization, and user interface.

Woebot and Happify users reported several limitations and areas for improvement during the first month of the intervention (Table 7). One of the most significant drawbacks was that both applications supported only English, which could be a major barrier for non–English speaking users. This lack of language diversity highlights the need for mental health apps that cater to a wider range of users.

Another common issue was the repetitive and limited nature of the responses provided by the apps. Despite Woebot’s diverse content, users noted that certain parts felt repetitive. Happify users also reported a lack of content variety. This suggests that even with advanced conversational agents, the interactions can still feel scripted and constrained by rule-based systems.

Woebot users encountered problems with the user input system, which sometimes led to infinite loops or errors in the conversation flow. This could be frustrating for users seeking smooth and intuitive interactions. Happify users also found the interface less intuitive than expected.

In terms of conversational abilities, both apps had limitations. Woebot’s responses could feel fixed and repetitive, while Happify’s questions were sometimes perceived as repetitive. These issues underscore the challenges in creating truly dynamic and engaging conversational experiences.

The attrition rates were notably different between the 2 intervention groups. The Woebot group saw a decrease from 22 to 15 participants, whereas the Happify group saw a decrease from 21 to 9 participants. This significant attrition in the Happify group suggests that user satisfaction may have played a role in retention rates. Participants’ feedback indicated that issues such as nonintuitive design and pressure for premium subscription might have contributed to the higher dropout rate in the Happify group compared to the Woebot group.

Overall, users expressed a need for more relevant and applicable content in both apps. Woebot users desired content that was better tailored to their specific needs, while Happify users reported dissatisfaction with the overall experience and felt pressured to subscribe to premium services.

Table 8 summarizes the participants’ suggestions for enhancing the apps, which include improving the chatbot’s conversational abilities, providing more personalized content, offering a wider variety of interactive features, and enhancing the user interface. Participants also emphasized the importance of integrating these apps with professional mental health support systems.

These findings highlight the need for designing mental health apps that are linguistically inclusive, offer diverse and engaging content and intuitive user experiences, and enable effective interactions. Addressing these areas for improvement could greatly enhance the effectiveness and user satisfaction of digital mental health interventions. Moreover, this feedback underscores the essential need to integrate digital platforms with both web-based and offline medical systems, ensuring that students have seamless access to professional support. As Lambert and Barley [34] highlighted, therapeutic relationship is crucial for psychotherapy’s effectiveness, and our study’s participants similarly emphasized the importance of connecting with mental health professionals for effective digital applications.

**Table 6:** Participants’ favored features.

Category	Woebot (n=17)	Happify (n=9)
User experience	Conversational feel, daily journal, and warm messages	Well-chosen name and versatility
Contents	Gratitude journal and psychological knowledge	Thank activity and guided meditation
Features	Fast response, varied content, and cute images	Thank activity, guided meditation, and word game

**Table 7:** Areas for improvement.

Category	Woebot	Happify
Language support	English only	English only
Content variety	Variety but some repetition	Content seems a bit lacking
User experience	User input system or infinite loop problem	Nonintuitive and occurrence of errors
Conversational features	Fixed responses	Repetitive questions
Overall feedback	Desire for more applicable contents	Not satisfactory and pressure to subscribe

**Table 8:** App enhancement suggestions (n=31).

Suggested enhancement	Mentions, n (%)
Inclusion of a system for connecting with mental health professionals	12 (39)
Reward system for activities performed	7 (23)
High-quality feedback for gratitude journals	5 (16)
Home button and feature descriptions on pages	4 (13)
Alarm system for activities to be performed	4 (13)
Personalized opt-in function based on individual needs	4 (13)
Various video content for users	3 (10)
Podcast on loneliness and depression	2 (6)
UCC^a^ and peer-to-peer networking	2 (6)

^a^UCC: user-created content.

#### Coping Mechanisms for Loneliness

During the focus group interviews, participants shared a diverse array of coping mechanisms for loneliness (Textbox 2). These strategies encompassed goal-setting, distraction, self-care, and social connection.

Participants emphasized redirecting focus toward tangible achievements and using diverse forms of distraction to manage loneliness. Self-care activities and interpersonal engagement were highlighted as crucial aspects of coping with loneliness. These insights suggest that a multifaceted approach addressing individual coping strategies and broader social connections is essential.

**Coping strategies for loneliness.**

**Goal-setting**

- Participants emphasized redirecting their focus toward tangible achievements, such as studying for English proficiency certificates or scholarships. “I’m motivated to achieve things. I like to find a way out and do various things. After graduating from high school, I started reading books seriously, even though I avoided classic novels like Frankenstein because they were difficult.”

**Distraction**

- Overcoming emotional entanglement, participants turned to diverse forms of distraction. “I used to get caught up in my emotions, but lately, I’ve been trying to get out of them. For example, I watch interesting TV shows. I listen to new songs, both upbeat and ballads. I find something to do to distract myself. I watch TV or YouTube. I find something I can immerse myself in. I watch games or dramas. I watched ‘Iltascansdal.’ I watch games or dramas.”

**Self-care**

- Self-care strategies include going for walks, reading specific books, and consciously managing negative thoughts. “Life is a journey, so it’s important to be strong on your own. I’ve been working out at the gym recently.” Participants emphasized regular exercise routines, waking up on time, and engaging in activities such as writing during challenging times.

**Social connection**

- Interpersonal engagement emerged as a key coping mechanism, with participants highlighting the significance of talking to people, especially close friends, to alleviate loneliness. “I’ve tried various things, but talking to people is the only way to forget those feelings. I prefer to talk to close friends rather than new people. I miss being around people. I cope by talking to other people. I talk to other people and express my feelings.”

#### Gratitude Journaling and Positive Experiences

Participants’ responses to the gratitude journaling feature of the apps revealed instances of positive emotional transformation. Engaging in gratitude journaling enhanced self-compassion, fostered acceptance, and strengthened social bonds. The interactive nature of the chatbots and the sense of care conveyed through warm greeting messages were particularly appreciated by participants dealing with loneliness or isolation.

The word cloud in Multimedia Appendix 9 visually represents participants’ positive experiences with the app, based on their responses to questions about specific moments they found helpful. The word size represents the frequency of mentions, with “gratitude,” “daily,” “conversation,” “meditation,” “response,” and “writing” being the most common words. This indicates that participants frequently benefited from the app in daily gratitude practices, meditation, conversations, and writing. The word cloud clearly highlights these common themes and experiences.

Table 9 presents the specific instances when participants found the apps most beneficial, categorized by the positive changes they experienced, such as emotion enhancement, gratitude journaling, daily life improvement, and physical health improvement. Participants reported increased happiness, reduced negative emotions, better sleep quality, enhanced concentration, and even physical health benefits such as reduced blood pressure and fatigue through app use.

The examples in Table 9 suggest that digital mental health interventions have the potential to positively impact various aspects of college students’ well-being, even if these effects were not fully captured in the quantitative analysis. The qualitative data indicates that consistent use of these apps could lead to meaningful improvements in users’ mental health and overall well-being.

**Table 9:** Specific examples of best used cases.

Category and subcategory		App	Positive change	Specific example
**Gratitude journal**				
	Positive emotion enhancement	Woebot and Happify	Increased happiness and sense of accomplishment, reduced negative emotions, and the development of a positive mindset	“When I wrote down what I was grateful for every day in Woebot, I was able to be grateful for even the small things.” “Through Happify’s Thank You, there were times during the week when I thought about the things I was grateful for and felt happy.”
	Self-reflection	Woebot and Happify	Reflecting on the day, understanding oneself, and exploring positive values	“When I used the journal to write down three things I was grateful for each day in Woebot, I was able to reflect on the day.” “Writing a gratitude journal in Happify made me think about the things I was grateful for.”
	Daily life improvement	Woebot and Happify	Reduced stress, increased mental stability, and improved sleep quality	“When I felt really frustrated, writing a gratitude journal in Woebot helped me to feel neutral.” “When I tried to submit a screenshot of my gratitude journal in Happify, I was surprised to see the old gratitude I had forgotten.”
**Meditation and deep breathing**				
	Concentration improvement	Happify	Improved concentration, reduced distractions, and efficient time management for goal achievement	“On the way home on the subway, or in bed before going to sleep, I wrote thank activity, and I was able to look back on the day and feel relieved that I was able to get through the day safely, and that there were things to be grateful for, even if they were small.”
	Physical health improvement	Woebot	Reduced blood pressure and heart rate, increased immunity, relaxed muscles and relieved pain, improved sleep quality, and reduced fatigue	Brief meditation before sleep for emotional regulation: One participant mentioned, “Before going to bed, I used the brief meditation, which helped me organize my emotions and feel more at ease.”
	Emotion regulation	Woebot	Reduced anxiety, stress, and depression; improved emotion recognition and regulation ability; and maintenance of peace of mind	“There was a moment when I was able to regulate my emotions.” “One day, when someone I was in touch with said they were going to confess, I suddenly thought of the app and wrote that I was grateful.”
**Miscellaneous**				
	Positive emotions	Bondee	Emotional stability and healing effect	“The experience of just floating sky photos when I’m tired.”

## Discussion

### Principal Findings

This study adds to the research on digital mental health interventions by evaluating the effectiveness of Woebot and Happify in reducing loneliness and depression among college students. Compared to the study by Fitzpatrick et al [26], our study had a longer intervention period and a postintervention follow-up for a more comprehensive evaluation. While the study by Lim et al [24] had a similar intervention period, our study had a larger sample size and more extensive analyses. Unlike Boucher et al [25], who focused on qualitative insights during a shorter period, our mixed methods approach that spanned a longer duration provided a better understanding of the interventions’ efficacy and user experiences. Our study uniquely compared Woebot and Happify, offering insights into their effectiveness and user preferences.

The quantitative data showed a moderate positive correlation between loneliness and depression (*r*=0.37; *P*<.001), highlighting their complexity. This suggests that while related, loneliness and depression are distinct, and other factors may also influence college students’ mental health outcomes (see the PHQ-9 and UCLA Loneliness Trend Analysis code in Multimedia Appendix 5).

The moderate correlation between loneliness and depression highlights their intricate, bidirectional relationship. Loneliness can lead to depressive symptoms due to a lack of social connection, while depression can increase loneliness through social withdrawal. This suggests that interventions targeting either issue may positively affect the other. However, loneliness and depression are distinct constructs that require different approaches: social skills and connections for loneliness and CBT and emotion regulation for depression.

In addition, the moderate correlation suggests that other factors, such as stress, coping mechanisms, and resilience, also play a role in mental health outcomes. This underscores the need for comprehensive, multifaceted interventions that address the diverse challenges faced by college students. Even within the small control group using Bondee, the slight decrease in loneliness and depression scores suggests that any form of structured interaction can have beneficial effects, emphasizing the importance of social engagement and connection.

Several factors might explain why the interventions did not yield significant effects. First, the lack of a Korean version of the apps may have reduced engagement, especially for those less proficient in English.

In addition, the demanding academic schedule and competitive environment, coupled with daily stressors, may have exacerbated depression and loneliness, reducing intervention effectiveness. This underscores the need for holistic digital mental health interventions considering these factors.

### Additional Considerations for the South Korean Context

In South Korea, research on digital interventions for reducing loneliness and depression among college students is extremely rare. There are very few studies, and none involve structured intervention experiments. This highlights the importance of developing and validating Korean-language digital mental health interventions. Given the severe mental health crisis among Korean college students, immediate intervention is crucial.

South Korean college students highly valued conversational elements, positive reinforcement, educational features, and reward mechanisms in digital interventions. This study serves as a critical pilot for the future development of Korean-language support features as well as reward-based CBT chatbots integrating positive psychology and empathetic support. The outcomes of this research provide a foundational understanding that will guide the creation of more effective and culturally relevant digital mental health tools for South Korean college students.

### Implications

The findings of this study have several important implications for future research and practice in the field of digital mental health interventions for college students (Textbox 3).

**Implications of this study.**

**Implications**

1. Personalized, user-centered design: future interventions should be tailored to the unique needs and preferences of college students to enhance engagement and effectiveness. For example, conducting in-depth user interviews to develop customized opt-in features and various personas can significantly improve the personalization of digital mental health tools. As mentioned in
  Table 8
  , features such as alarm systems for activities and personalized opt-in functions based on individual needs were frequently suggested by participants.
2. Integration with professional and peer support: digital interventions should seamlessly connect with both web-based and offline mental health professionals. In addition, peer support groups, for example, the University of Michigan’s Wolverine Support Network [35] and listener systems such as 7 Cups [36], can be effective alternatives for providing emotional support and fostering a sense of community.
3. Mental health literacy education: incorporating mental health literacy education into college curricula and developing holistic support systems is crucial for addressing the persistent challenges faced by students. Institutions should prioritize comprehensive approaches to mental health promotion.
4. Longitudinal research: further research with larger, more diverse samples and extended follow-up periods is needed to validate the efficacy and long-term impact of digital interventions. This will help in understanding their sustained benefits and potential limitations.
5. Development of large language model–based chatbots: enhancing chatbots with Korean-language support, positive psychology, empathy, and reward mechanisms can potentially better meet the mental health needs of South Korean college students, improving engagement and effectiveness. Future research should focus on developing and evaluating such chatbots tailored to the specific needs and preferences of South Korean college students.

### Limitations

This study has several limitations that should be acknowledged. First, the small and unbalanced sample sizes across the study groups may have limited the statistical power and generalizability of the results. The notably smaller control group compared to the intervention groups could have influenced the nonsignificant findings. In addition, due to the limited sample size and the need to ensure sufficient power in the intervention groups, an uneven distribution of depression scores was observed at baseline (*P*=.78). Although the mean depression scores were within a similar range across the groups, this limitation should be considered when interpreting the results, as the groups may not be perfectly comparable in terms of initial depression severity. Future studies with larger and more balanced samples are needed to confirm the efficacy of digital interventions for college students.

Second, the reliance on self-report measures may have introduced response bias. Incorporating objective measures and diagnostic assessments could enhance the validity of future studies. Third, the use of English-language interventions may have influenced engagement and satisfaction among participants with lower English proficiency. Future research should explore the development and evaluation of interventions in participants’ native languages. Finally, the study’s duration may have been insufficient to capture long-term effects. Longer follow-up periods are needed to assess the sustainability of treatment effects and identify potential barriers to long-term engagement with digital mental health tools.

### Conclusions

This mixed methods study provides valuable insights into the efficacy and user experiences of digital mental health interventions for college students. While the quantitative findings did not show significant effects, the qualitative data revealed the potential of well-designed digital interventions to positively impact various aspects of college students’ mental health and well-being. Participants’ experiences with gratitude journaling, meditation, and other app features suggest that consistent use of these tools could lead to meaningful improvements in emotional, cognitive, and physical health.

However, the study also underscores the importance of considering contextual factors and individual preferences when designing and implementing digital mental health interventions. Future research should focus on developing personalized, user-centered interventions that address the unique needs of college students and explore ways to integrate these tools with professional support systems. Moreover, a comprehensive approach to promoting mental health among college students is needed, which includes mental health education and the provision of holistic support systems. Further large-scale, longitudinal studies are necessary to confirm the efficacy and sustained impact of digital interventions on college students’ mental health outcomes.

## Appendix

Multimedia Appendix 1 Participants instruction manual (in Korean).

Multimedia Appendix 2 Recruitment notice and survey questionnaires (in Korean).

Multimedia Appendix 3 Depression and University of California, Los Angeles Loneliness scale questionnaires (self-reported, in Korean).

Multimedia Appendix 4 Focus group interview orientation (in Korean).

Multimedia Appendix 5 Data analysis code.

Multimedia Appendix 6 Institutional review board approval letter.

Multimedia Appendix 7 Informed consent form (in Korean).

Multimedia Appendix 8 CONSORT-EHEALTH (Consolidated Standards of Reporting Trials of Electronic and Mobile Health Applications and Online Telehealth) checklist (in English).

Multimedia Appendix 9 Word cloud showcasing students’ positive experiment experiences.

## Abbreviations

CBT: cognitive behavioral therapy
CONSORT: Consolidated Standards of Reporting Trials
CONSORT-EHEALTH: Consolidated Standards of Reporting Trials of Electronic and Mobile Health Applications and Online Telehealth
IRB: institutional review board
PHQ-9: Patient Health Questionnaire-9
UCLA: University of California, Los Angeles
