# Supplementary material for: Digital Interventions for Reducing Loneliness and Depression in Korean College Students: Mixed Methods Evaluation
Source: JMIR Form Res. 2024 Sep 12;8:e58791. doi: 10.2196/58791 (PMC11427852; doi:10.2196/58791)

MULTIMEDIA APPENDIX (1)

1. Participants Instruction Manual (in Korean)

This document provides instructions for participants in the study, including how to download and use the mental health chatbot applications (Woebot and Happify) and the control group application (Bondee). Participants are required to use the assigned application for at least 15 minutes daily and complete specific tasks within the applications. Participants are also instructed to capture and save screenshots of their activity as a record of their participation, which will be used for participant compensation.

1-1. Woebot 실험 참가자 안내 사항

안녕하세요? 여러분은 연구동의서에 설명되어 있듯이 실험에 자발적으로 참여하셨고 정신건강챗봇인 woebot 에 랜덤으로 배정되셨습니다.

Woebot 은 기본적으로 CBT(Cognitive Behaviour Therapy) 기반 챗봇으로 의사의 처방을 수반한지 않아도 사용가능한 디지털 어플리케이션으로 기본적인 임상적 유효성이 2017 년 6 월에 JMIR Mental Health 저널에 출판되었습니다 (http://mental.jmir.org/2017/2/e19/).

Woebot 은 구글 플레이와 애플 앱스토어어에서 다운로드 받으실 수 있습니다.

구글플레이: https://play.google.com/store/apps/details?id=com.woebot&hl=en&gl=US

앱스토어: https://apps.apple.com/us/app/woebot-your-self-care-expert/id1305375832

실험 참가자 준수 사항

1)앱을 다운 받으십시오

2) 계정을 만드세요 이메일과 패스워드 생년월일, 닉네임을 입력하게 되어 있습니다

3) 매일 최소 15 분간 앱을 사용하셔야 합니다.

4) 15 분 동안 토픽(아래 그림 참조) 중에서 최소 4 개의 토픽, (Focusing on positive, Relationship, Mindfulness & Meditation, Managing emotions 필수) 과 저널(Gratitude Journal 필수)을 사용을 사용하시면 됩니다.

5) 사용기록을 위해 Gratitude Journal 을 주 3 회 캡처해서 보관하시기 바랍니다. 매월 실험 참가자 보상을 위해 필요하니 꼭 보관하시기 바랍니다.

<어플리케이션 화면 예시>

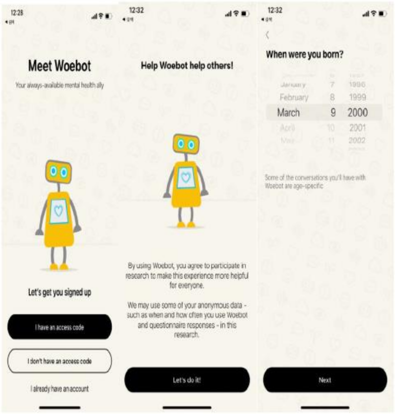

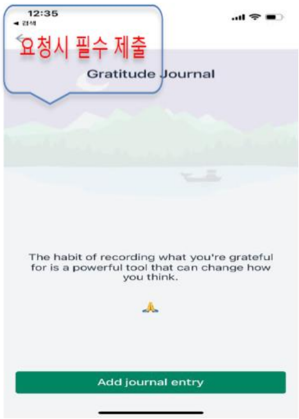

1-2. Happify 실험 참가자 안내 사항

안녕하세요? 여러분은 연구동의서에 설명되어 있듯이 실험에 자발적으로 참여하셨고 정신건강챗봇인 Happify 에 랜덤으로 배정되셨습니다.

Happify 는 기본적으로 CBT(Cognitive Behavior Therapy) 기반 챗봇으로 의사의 처방을 수반한지 않아도 사용 가능한 디지털 어플리케이션으로 그 효과성이 JMIR Mental Health 저널에 출판되었습니다. 최근 출간된 논문을 공유합니다. https://mental.jmir.org/2021/2/e26617/

Happify 는는 구글 플레이와 애플 앱스토어어에서 다운로드 받으실 수 있습니다.

구글플레이: https://play.google.com/store/apps/details?id=com.happify.happifyinc&hl=en&gl=US

앱스토어: https://apps.apple.com/us/app/happify-for-stress-worry/id730601963

실험 참가자 준수 사항

1)앱을 다운 받으십시오

2) 계정을 만드세요 성별, 나이, 인종, 직업, 가족관계 및 현재 심리상태 등의 정보를 입력하게 되어 있습니다

3) 매일 최소 15 분간 앱을 사용하셔야 합니다.

4) 앱은 크게 인스턴트 플레이(Instant Play)와 트랙(무료, 유료) 트랙이 있는데 15 분 동안 Instant Play (아래 그림 참조) 중에서 최소 3 개의 activity(Serenity Scene, Guided Meditation, Negative Knockout), 그리고 그 activity 에서 Thank mode 는 필수) 과 Mindfulness & Meditation (무료 트랙)을 사용하시면 됩니다.

5) 사용기록을 위해 activity task 중 Thank mode(뒤에 화면 예시) 주 3 회 캡처하거사 업로드해서 보관하시기 바랍니다. 매월 실험 참가자 보상을 위해 필요하니 꼭 보관하시기 바랍니다.

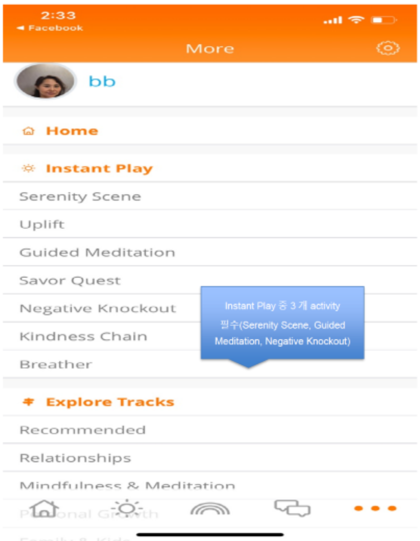

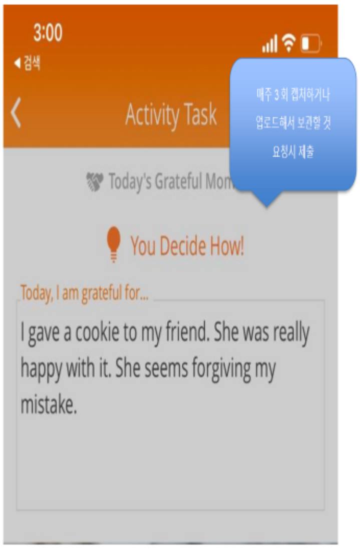

1-3. 대조군 Bondee (안내 사항)

여러분은 연구동의서에 설명되어 있듯이 실험에 자발적으로 참여하셨고 메타버스 SNS 인 Bondee 어플리케이션션에 랜덤으로 배정되셨습니다. Bondee(본디)는 2022 년말 출시 이래 사람들 사이에서 빠르게 퍼져나간 메신저 기반 메타버스 앱으로 홈 화면에서는 내 상태를 다양한 상태로 설정할 수 있습니다. 그리고 사용자가 상태를 변경할 때마다 친구들이'좋아요'나 댓글도 남길 수 있으며 실시간으로 사진을 찍어 아바타 위에 띄울 수도 있습니다.

Bondee 는 구글 플레이와 애플 앱스토어어에서 다운로드 받으실 수 있습니다.반드시 단말기 설정을 영어로 해놓고 **영어버전**으로 다운 받으시고 실험 규정상(다른 어플리케이션과 다른 사용자와의 형평상) 영어로사용하시기를 바랍니다.

구글플레이: https://play.google.com/store/apps/details?id=com.metadream.bondee&hl=en&gl=US

앱스토어: https://apps.apple.com/my/app/bondee/id6443947003

실험 참가자 준수 사항
1)앱을 다운 받으십시오
2) 계정을 만드세요
3) 매일 최소 15 분간 앱을 사용하셔야 합니다.
4) 15 분 동안의 사용 기능(집꾸미기, 플로팅, 채팅) 제한은 없으나 친구를 3 명 이상 초대해서서로 채팅 및 인터랙션 하시기를 추천합니다
5) 사용기록을 위해 단말기 화면을 주 3 회 캡처해서 보관하시기 바랍니다. 매월 실험 참가자 보상을 위해 필요하니 꼭 보관하시기 바랍니다.

<본디 예시 화면>

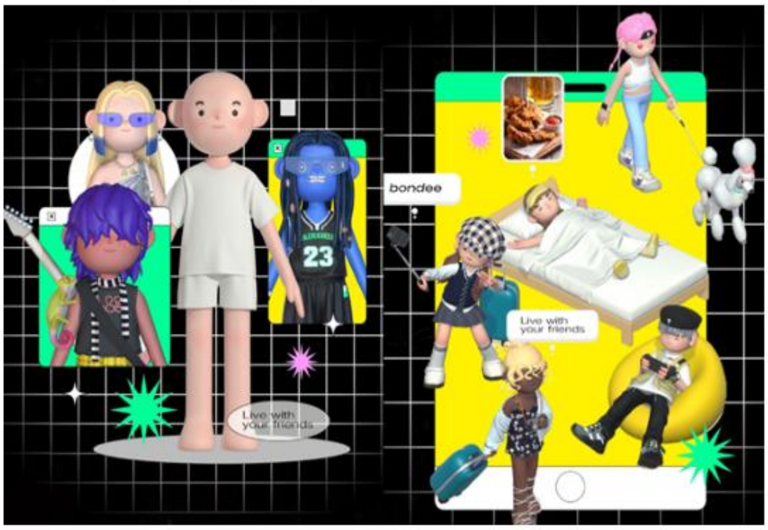

Supplement: Multimedia Appendix 1 [file formative_v8i1e58791_app1.pdf]
