# Supplementary material for: Digital Interventions for Reducing Loneliness and Depression in Korean College Students: Mixed Methods Evaluation
Source: JMIR Form Res. 2024 Sep 12;8:e58791. doi: 10.2196/58791 (PMC11427852; doi:10.2196/58791)
Supplement: Multimedia Appendix 2 [file formative_v8i1e58791_app2.pdf]

MULTIMEDIA APPENDIX (2)

2. Recruitment Notice and Survey Questionnaires (in Korean)

2-1. Recruit: (February 28, 2023: 1st recruit, March 8, 2024: 2nd recruit)
https://www.skku.edu/skku/campus/skk_comm/notice01.do?mode=list&srCategoryId1=20&srSearchKey=article_title&srSearchVal=%EB%8B%B9%EC%8B%A0%EC%9D%80+%ED%98%84%EC%9E%AC

2-2. Baseline Survey (February 28~March 10)
https://forms.gle/ryeY22vbYVUageCz5

2-3. 1st intervention survey (April 7~April 15)
https://forms.gle/vbtpvds9dh9nZ7fo9

2-4. 2nd intervention survey (May 15 ~ May20)
https://forms.gle/QCuLvZRKrFGhRCLV7

2-5. Post intervention survey (June 15~ June 20)
https://forms.gle/6prkAictWC25MVdK8
