# Supplementary material for: Digital Interventions for Reducing Loneliness and Depression in Korean College Students: Mixed Methods Evaluation
Source: JMIR Form Res. 2024 Sep 12;8:e58791. doi: 10.2196/58791 (PMC11427852; doi:10.2196/58791)
Supplement: Multimedia Appendix 3 [file formative_v8i1e58791_app3.pdf]

MULTIMEDIA APPENDIX (3)

3. Depression and UCLA Loneliness Scale Questionnaires (Self-reported, in Korean)

The PHQ-9 (Patient Health Questionnaire-9) and UCLA Loneliness Scale Version 3 (Korean version) were included in the following four online surveys conducted via Google Forms:

① PHQ-9 (Patient Health Questionnaire-9): A depression screening tool

우울증 및 UCLA 외로움 지수 측정 설문지(자기보고형)

한글판 우울증 선별도구(Patient Health Questionnaire-9)

본 검사는 우울한 정도를 스스로 알아보기 위한 것입니다. 이 질문들이 확정된 진단을 위한 것은 아니지만 높은 점수가 나왔을 경우에는 우울증의 가능성이 높으므로, 더 정확한 평가를 위해서 병원에서 진료를 받아볼 것을 추천합니다.

지난 2주간, 얼마나 자주 다음과 같은 문제들로 곤란을 겪으셨습니까?
지난 2주 동안에 아래와 같은 생각을 한 날을 헤아려서 해당하는 숫자에 표시하세요.

지난 2주 동안에	없음	2, 3일 이상	7일 이상	거의 매일
1. 기분이 가라앉거나, 우울하거나, 희망이 없다고 느꼈다	0	1	2	3
2. 평소 하던 일에 대한 흥미가 없어지거나 즐거움을 느끼지 못했다	0	1	2	3
3. 잠들기가 어렵거나 자주 깼다/혹은 너무 많이 잤다	0	1	2	3
4. 평소보다 식욕이 줄었다/혹은 평소보다 많이 먹었다	0	1	2	3
5. 다른 사람들이 눈치 챌 정도로 평소보다 말과 행동이 느려졌다/ 혹은 너무 안절부절 못해서 가만히 앉아있을 수 없었다	0	1	2	3
6. 피곤하고 기운이 없었다	0	1	2	3
7. 내가 잘못 했거나, 실패했다는 생각이 들었다/ 혹은 자신과 가족을 실망시켰다고 생각했다	0	1	2	3
8. 신문을 읽거나 TV를 보는 것과 같은 일상적인 일에도 집중할 수가 없었다	0	1	2	3
9. 차라리 죽는 것이 더 낫겠다고 생각했다/혹은 자해할 생각을 했다	0	1	2	3

② UCLA Loneliness Scale Version 3, Korean version

	문 항	전혀 아니다	드물지만 있다	가끔 있다	항상 그렇다
1	얼마나 자주 주변 사람들과 잘 통합니까?				
2	얼마나 자주 사람들과의 교제가 부족하다고 느낍니까?				
3	얼마나 자주 도움을 청할 사람이 아무도 없다고 느낍니까?				
4	얼마나 자주 혼자라고 느낍니까?				
5	얼마나 자주 친구들 모임에 속해 있다고 느낍니까?				
6	얼마나 자주 당신 주위 사람들과 공통의 관심사를 가지고 있습니까?				
7	얼마나 자주 당신이 더 이상 아무하고도 가깝지 않다고 느낍니까?				
8	얼마나 자주 당신의 흥미와 생각들이 주변사람과 나누어지지 않는다고 느낍니까?				
9	얼마나 자주 자신이 외향적이고 우호적이라고 느낍니까?				
10	얼마나 자주 사람들과 가깝다고 느낍니까?				
11	얼마나 자주 혼자 남겨졌다고 느낍니까?				
12	얼마나 자주 다른 사람들과의 관계가 의미 없다고 느낍니까?				
13	얼마나 자주 당신을 진정으로 아는 사람이 아무도 없다고 느낍니까?				
14	얼마나 자주 다른 사람들로부터 고립되어 있다고 느낍니까?				
15	얼마나 자주 당신이 원할 때 친구들을 사귈 수 있다고 느낍니까?				
16	얼마나 자주 당신을 진정으로 이해해주는 사람들이 있다고 느낍니까?				
17	얼마나 자주 수줍음을 느낍니까?				
18	얼마나 자주 사람들이 당신과 진정으로 함께 있지 않고 그저 주위에 있는 것이라고 느낍니까?				
19	얼마나 자주 당신과 얘기를 나눌 사람들이 있다고 생각합니까?				
20	얼마나 자주 당신이 도움을 청할 수 있는 사람들이 있다고 느낍니까?				
