# Supplementary material for: Digital Interventions for Reducing Loneliness and Depression in Korean College Students: Mixed Methods Evaluation
Source: JMIR Form Res. 2024 Sep 12;8:e58791. doi: 10.2196/58791 (PMC11427852; doi:10.2196/58791)
Supplement: Multimedia Appendix 4 [file formative_v8i1e58791_app4.pdf]

MULTIMEDIA APPENDIX (4)

4.Focus Group Interview Orientation

The document provided to participants prior to the focus group interviews, introducing the concept of focus group interviews and their methodology. It also included a list of anticipated questions, covering topics such as experiences of loneliness, coping mechanisms, and feedback on the apps used in the study. However, due to time constraints, only a subset of these questions were addressed during the actual interviews, primarily focusing on coping with loneliness and user experiences with the apps (Bondee, Happify, Woebot).

The key questions discussed in the interviews were:

- How do you cope with loneliness? What do you do when you feel lonely?
- Please share your thoughts and experiences as a user of the (Bondee, Happify, Woebot) app over the past month.
- In what ways did the app help you the most?
- What aspects of the app do you think could be improved?

The full text of the orientation document is available at Focus_Group_Interview_Orientation(in Korean)
