# Supplementary material for: Digital Interventions for Reducing Loneliness and Depression in Korean College Students: Mixed Methods Evaluation
Source: JMIR Form Res. 2024 Sep 12;8:e58791. doi: 10.2196/58791 (PMC11427852; doi:10.2196/58791)
Supplement: Multimedia Appendix 5 [file formative_v8i1e58791_app5.pdf]

MULTIMEDIA APPENDIX (5)

5. Data Analysis Code

The data analysis for this study was conducted using Python in Google Colab, a cloud-based Jupyter notebook environment. If you have any questions about the code or encounter issues accessing the notebook, please contact the corresponding author.

5-1. PHQ9 & Loneliness trend Analysis

PHQ9&UCLA_Loneliness_Trend code.ipynb

5-2. Cronbach analysis

baseline_cronbach.ipynb
1month_cronbach.ipynb
2month_cronbach.ipynb
post_cronbach.ipynb

5-3. Behavior Belief(Hypotheses Analysis)

(sum)behavior belief.ipynb

5-4. Self-report Mental health literacy

Self-report_literacy.ipynb

***** Data Availability Statement**

The anonymized data on participants' loneliness and depression measures collected throughout the intervention are available upon reasonable request to the corresponding author. The data will be provided in a deidentified format to protect participants' privacy and confidentiality. Requestors will be required to sign a data sharing agreement that specifies the terms of use and prohibits any attempts to reidentify participants.

If applicable, the data sets generated and analyzed during the current study are available in a publicly accessible : [Intervention_Data_#58791]. Additionally, the code used for analysis is available as above.

If data access is restricted, it is due to [reason for restriction, e.g., privacy concerns, institutional policy]. Interested researchers may contact the corresponding author to request access. The request process includes [describe steps for requesting access, e.g., submission of a data use agreement, review by an ethics committee].
