# Supplementary material for: Digital Interventions for Reducing Loneliness and Depression in Korean College Students: Mixed Methods Evaluation
Source: JMIR Form Res. 2024 Sep 12;8:e58791. doi: 10.2196/58791 (PMC11427852; doi:10.2196/58791)
Supplement: Multimedia Appendix 6 [file formative_v8i1e58791_app6.pdf]

MULTIMEDIA APPENDIX (6)

6. SKKU IRB Approval Letter

NOTIFICATION LETTER OF SKKU IRB

The document Retention Period is 3 years			
Institution	**Sungkyunkwan University**		
Research Director	BOYOUNG KANG		
IRB File NO.	SKKU 2023-02-043	Notification date	2023. 02. 27.
Title(Korean)	**자연어 처리 기술을 이용한 근거 기반 청년 정신건강 돌봄 디지털 서비스 플랫폼 개발**		
Title(English)	Evidence based young adults depression cohorts digital healthcare service platform		

Research Classification 1	▣ Human Object ☐ Human remains ☐ Embryos					
	☐ Somatic cell cloning embryos ☐ Embryonic stem cell stock					
	☐ etc					
Research Classification 2	▣ Survey ☐ Observation ☐ Data Analysis ▣ Arbitration					
	☐ Behavior ☐ Cohort ☐ Clinical trial					
	☐ etc					
Research Classification 3	◉ Prospective Research ○ Retrospective Research ○ Parallel					
Research Classification 4	☐ Non-clinical study(in vitro, in vivo preclinical study)					
Subjects to experiment	Total	60	Domestic	60	SKKU	60
Approval period	2023.02.27. ~ 2024.02.26					

Approval date	2023. 02. 27.
Interim Report date	2023. 12. 26.
Result	◉ Approval ○ Correctional approval ○ IRB review exemption

Issued by

IRB President : Prof. Kim, Ho Joong (M.D. Ph.D)

Institution : **Sungkyunkwan University** (*SKKU)
Address : **#2066, Seobu-ro, Jangan-Gu, Suwon, Korea, 16419**
IRB President : Prof. Kim, Ho Joong (M.D. Ph.D)
Dept. of Pulmonary and Critical Care Medicine, Samsung Medical Center(*SMC)
