# Supplementary material for: Digital Interventions for Reducing Loneliness and Depression in Korean College Students: Mixed Methods Evaluation
Source: JMIR Form Res. 2024 Sep 12;8:e58791. doi: 10.2196/58791 (PMC11427852; doi:10.2196/58791)
Supplement: Multimedia Appendix 7 [file formative_v8i1e58791_app7.pdf]

MULTIMEDIA APPENDIX (7)

7.Informed Consent Form (in Korean)

The informed consent form was provided to all participants before their enrollment in the study. The form is written in Korean. The consent form includes information about the purpose of the study, which is to investigate the prevalence of loneliness and depression among young adults and to explore the effectiveness of digital interventions. It also outlines the study procedures, including the use of mental health apps (Woebot and Happify) or a recently launched SNS app (Bondee), monthly online surveys, and focus group interviews. The form explains the risks and benefits of participation, confidentiality measures, and the voluntary nature of the study.

Participants were required to sign the consent form, indicating their understanding of the study and their willingness to participate.

[성균관대 기관생명윤리위원회(IRB)]

연구참여자 동의서 (심층면접 연구용)

연구과제	국문	자연어 처리 기술을 이용한 근거 기반 청년 정신건강 돌봄 디지털서비스 플랫폼 개발					
	영문	Evidence based young adults depression cohorts digital healthcare service platform					
연구자		소속	직위	성명	전공분야	연락처	
						전화번호	C.P.
	연구책임자	성균관대학교 산학협력단	박사과정 학생(연구 등록학기)	강보영	융합전공		
	연구담당자	성균관대학교	교수	홍문표	융합전공교수		

※. 본 연구의 연구자는 귀하에게 심층 면접에 참여를 부탁드리고자 합니다. 아래의 설명을 잘 읽으시고 의문사항이 있으시면 위 연구자에게 질문하여 주시기 바랍니다.

1. 연구 배경

본 연구의 목적은 청년 외로움· 우울증 실태 조사 및 디지털 인터벤션의 효과 탐색 연구하기 위한 목적으로 수행됩니다.

사업명: 보건복지부 K-Medi융합인재양성지원사업
과제명: 자연어 처리 기술을 이용한 근거 기반 청년 정신건강 돌봄 디지털 서비스 플랫폼 개발
주관기관: 성균관대학교 산학협력단
연구개발 과제번호: HI22C2185
연구책임자:강보영

■ 실험 목적
- 우울증 및 정신 건강의 문제를 야기할 수 있는 청년들의 외로움을 감소시키고 정신 건강을 돕기 위한 어플리케이션(woebot, happify) 및 최근 출시된 SNS 앱을 사용한 후의 피험자의 외로움, 우울과 관련한 정신건강 바로미터를 측정한 정량적 데이터 및 사용한 후기의 정성적 데이터를 분석.
■ 인원 : 30명 (인문사회캠퍼스), 30명(자연과학캠퍼스)

2. 연구방법

- 실험 장소: 비대면
- 1개월에 1회 온라인 링크로 접속하여 자가설문지 응답(모든 데이터는 익명화 처리).
- 매일 회당 15분 이상 정도의 어플리케이션 사용(자세한 사용방법은 이메일 개별 공지 혹은 실시간 Q&A 채팅)
- 월 1회(월 둘째 주 시간 나중에 공지) 실시간 온라인 비대면으로 포커스 그룹 인터뷰(FGI) 약 60 분간 진행,
- 월 1회 (월 둘째 주 외로움 지수 & 우울 지수 측정 설문)

한 그룹당 4~5명 정도 귀하의 경험을 충분히 이야기하는 심층면담으로 진행됩니다. 1번에 걸리는 시간은 60 분 정도이며, 부족한 부분이 있으면 귀하의 동의하에 추가로 1-2 번의 인터뷰를 더 하게 되어 모두 2-3회를 하게 됩니다. 인터뷰 내용은 녹음될 것이며, 필사본으로 전환되어 분석되게 됩니다.

질문의 예는 다음과 같으며, 귀하의 답변을 듣고 추가적인 질문을 하게 됩니다.

(질문의 예)

- 본 실험에 처음 참여했을 때 다른 사람들과 더 연결되고 싶다(싶지 않다)고 언급했었습니다. 더 연결되(지 않)길 바란 이유는 무엇이었나요?
- 여러분은 외로움을 느끼게 하는 요인은 무엇인가요(어떨 때 외로우신가요?)
- 외로움을 느낄 때 어떻게 대처하시나요?
- 최근 외로움을 느낀 경험과 그 이유에 대해 이야기해 주실 수 있나요? 언제 있었나요?
- 외로움을 느낄 때 소속 학과, 학교, 여러분이 속한 사회의 지원 시스템이 있습니까? 어떤 역할을 하나요?
- 코로나 기간동안에 본인의 마음과 심리 상태의 변화가 있었습니까? 있었다면 어떠했는지 설명해주세요.
- 코로나 기간동안에 힘든 것이 있었다면 무엇이고 그중 가장 어떤 것이 힘들었나요?
- (우봇이나 해피파이, 본디)의 앱에서 본인이 가장 좋았던 두 가지 기능(절차와 콘텐츠 면에서)은 무엇입니까?
- (우봇이나 해피파이, 본디)의 앱을 사용함에 있어서 불편한 두 가지 점(절차와 콘텐츠 면에서)은 무엇입니까?
- (우봇이나 해피파이,본디)의 앱이 여러분에게 어떤 면에서 가장 도움이 되었습니까?
- (우봇이나 해피파이, 본디)의 앱을 사용함에 있어서 가장 어떤 점이 개선되면 좋겠습니까?
- (우봇이나 해피파이,본디)의 앱이 그런 방향으로 개선된다면 여러분의 외로움이나 정신 건강에 더 도움이 된다고 생각하시나요?
- 여러분은 앞으로 대학생활은 어떠하리라고 생각하나요?
- 본 실험에 참여하면서 여러분의 외로움, 정신건강 및 대학 생활에 대해서 하고 싶은 말이 있다면 자유롭게 얘기해주세요.

면담은 귀하가 편안하게 느끼는 안전한 장소에서 진행할 것이며, 자유롭고 솔직하게 경험을 있는 그대로 이야기 할 수 있도록 배려할 것입니다. 면담내용은 디지털 녹음기를 사용하여

녹음되며, 녹음 자료는 연구자만이 접근할 수 있는, 비밀번호가 설정된 개인용 컴퓨터에 안전하게 보관되었다가 연구가 종료되면 삭제될 것입니다.

3. 연구에 참여하여 얻게 되는 이득

본 연구에 참여함으로 해서 귀하에게 예상되는 직접적인 이득은 없습니다. 이 연구를 통하여 얻어진 지식은 모든 사람들이 이용할 수 있도록 학술 연구논문으로 출판될 수 있지만 귀하에게 직접적으로 이득이 제공되지는 않습니다. 다만 본 연구의 결과는 귀하와 같은 상황에 처한 분들을 도와드리는데 아주 귀중한 자료가 될 것으로 생각됩니다.

4. 개인정보에 대한 비밀보장

귀하가 말하신 내용은 녹음되고 문자로 입력될 것입니다. 그러나 귀하의 개인정보(이름, 생년월일, 거주지, 전화번호, 기타 누구인지를 알 수 있는 정보)는 가명으로 처리되어 누구의 경험이고 의견인지 모르게 할 것 입니다. 그리고 녹음 및 문자 내용은 연구 목적 외에는 절대로 사용되지 않을 것이며, 연구자만 접근할 수 있는 비밀번호가 설정된 개인용 컴퓨터에 안전하게 보관되었다가 연구가 종료되면 즉시 폐기될 것입니다. 연구 결과는 분석을 거쳐 자료제시의 형태로 (예를 들면 A씨는 다음과 같은 특징을 보였다) 발표될 것입니다.

5. 연구 참여에 따른 피해

연구 참여에 따른 큰 피해는 없을 것으로 예상됩니다. 그러나 장시간 인터뷰를 하는 도중 귀하가 겪은 경험들을 이야기하는 과정에서, 슬퍼지는 현상이 발생할 수 있습니다. 또한 뜻하지 않게 심하게 화가 나거나, 격한 감정이 나타날 수 있고, 우울증과 같은 심리적 어려움을 겪을 수도 있습니다. 본 연구자는 이러한 상황이 발생하지 않도록 최선을 다할 것입니다. 그럼에도 불구하고 피해가 발생했을 때는 다음과 같은 조치를 취할 것이며, 정신적 상처를 치료하기 위해 최선을 다하겠습니다.

1. 귀하가 인터뷰 도중에 격한 감정으로 더 이상 인터뷰를 할 수 없을 때, 언제든지 말씀하시면 인터뷰를 중지하거나 철회하도록 하겠습니다.
2. 인터뷰 도중 과도한 감정표출이 되지 않도록 최대한 배려하겠으며, 인터뷰는 한번에 60-90분을 넘지 않도록 하겠습니다. 만일 추가로 인터뷰가 필요한 경우에는 귀하의 동의를 받을 것이며, 귀하가 동의하시는 경우에만 추가로 1-2회 인터뷰를 실시하겠습니다.
3. 인터뷰 중이나 인터뷰 이후라도 인터뷰로 인한 귀하에게 심리적 문제가 발생할 때는, 본교의 상담전문가(이름과 전화번호를 적습니다)나 거주하시는 가까운 곳의 건강가정지원센터 또는 정신건강증진센터의 전문상담가를 통하여 심리적 상담을 받을 수 있도록 연결해 드리겠습니다. 만일 귀하가 다른 곳에서 상담받기 원하는 경우에는, 원하는 곳에서 상

담을 받으실 수 있습니다. 이때 비용은 참여자가 부담합니다.

6. 연구 준수사항

본 연구에 동의하시면, 연구를 위한 실험 및 심층면담을 진행하게 됩니다. 인터뷰 날짜와 시간은 연구 참여자와 상의하여 결정될 것입니다. 귀하의 경험에 대한 이야기가 본 연구의 가장 중요한 자료이므로 면담 시 성실히 이야기해 주시기를 부탁드립니다.

7. 연구기간 및 전체 연구 참여자 수

본 연구는 연구 승인 일로부터 4~6개월간 진행될 예정이며, 총 54명을 대상으로 시행할 예정입니다.

8. 연구에 참여해서 추가적으로 발생되는 비용은 없습니다.

9. 연구에 참여해서 직접적으로 지급되는 보상

본 연구에 참여하시면 응답율에 따라 사례비를 최대 20만원(설문지 1개당 1만원, 인터뷰 1회당 2만원, 실험참여 수당 최대 3만원, 연구종료시 추가보상(금액 미정))을 참여 횟수 및 이행률에 따라 차등 지급예정지급합니다. 사례비는 면담이 끝나고 매월말 정산해서 지급해드리겠습니다.

10. 자발적 참여 및 참여 거부와 철회의 자유

본 연구에 대한 참여는 전적으로 귀하의 의사에 따라 결정하시면 됩니다. 만약 본 연구에 참여하기를 원하지 않으시면 동의하지 않으셔도 되며, 또한 동의 후에도 마음이 바뀌는 경우, 언제라도 연구자에게 구두 또는 서면으로 참여 중지를 요청할 수 있습니다. 어떠한 선택을 하셔도 귀하에게 불이익이 없을 것입니다. 또한 중도 철회시 연구참여간 수집한 모든 정보는 즉시 파기하겠습니다.

11. 개인정보의 비밀 보장

본 연구의 결과 보고서나 출판물에서, 귀하의 개인정보는 비밀로 유지될 것이며 법에 따라 보호될 것입니다.

12. 자료의 추가사용에 대한 동의

연구가 잘 진행되면, 귀하의 자료는 향후의 다른 연구에서도 사용되어 더 좋은 결과를 이

루어낼 수 있습니다. 귀하의 자료를 향후의 다른 연구에 사용하는 것에 대해 다음 중 하나에 표시해 주시기 바랍니다.

☐ 향후의 모든 다른 연구에, 제 자료를 제공하여 연구하는 것에 동의합니다.

☐ 성균관대학교의 연구자에게만 동의합니다.

☐ 본 연구의 연구자에게만 동의합니다.

☐ 동의하지 않습니다.

13. 연구대상자로서의 권익에 관한 정보 제공

본 연구는 성균관대학교 기관윤리심의위원회(IRB)에 의해 검토되고 승인되었습니다. 만일 향후에 연구대상자의 권익에 피해를 주는 정보가 발생하는 경우, 연구자들은 조속한 시일 내에 알려드릴 것입니다. 본 연구에 대한 질문사항이 있으면 첫 페이지 상단의 연구자에게 문의하실 수 있으며, 연구참가자의 권리 침해에 대해 문의할 사항이 있으면 “성균관대학교 공동기기원 행정실 연구윤리 담당자 (TEL 031-299-6711)” 에 연락하실 수 있습니다.

14. 본 연구에 참가하기로 동의서에 서명하시면, 귀하는 서명된 동의서의 사본을 받게 될 것입니다.

본인은 본 동의서의 내용에 대해 설명을 들었고, 동의서 내용을 읽고 이해하였으며 본인이 궁금해하는 질문에 대한 답변을 들었습니다.

본인은 자발적으로 본 연구에 참여하는 것에 동의하므로 동의서에 서명합니다.

피험자

일자 20 년 월 일

성명 ____________________ (서명 또는 인)

※ 피험자가 제한능력자(미성년자 등)인 경우 법정대리인이 위에 성명기재 및 서명하고 제한능력자의 이름을 별도 기재함.

제한능력자(미성년자 등) 성명 / ____________

입회인(필요시)

일자 20 년 월 일

성명 ____________________ (서명 또는 인)

설명자 (책임연구자)

일자 20 23 년 3 월 10 일

성명 강보영
