# Supplementary material for: Digital Interventions for Reducing Loneliness and Depression in Korean College Students: Mixed Methods Evaluation
Source: JMIR Form Res. 2024 Sep 12;8:e58791. doi: 10.2196/58791 (PMC11427852; doi:10.2196/58791)
Supplement: Multimedia Appendix 8 [file formative_v8i1e58791_app8.pdf]

MULTIMEDIA APPENDIX (8)

8.CONSORT-EHEALTH Checklist

The CONSORT-EHEALTH checklist (V1.6) [34] was completed and is provided as a supplementary file. This checklist is an extension of the CONSORT statement, aimed at improving the reporting quality of eHealth trials.

**CONSORT-EHEALTH Checklist V1.6.2 Report**	**Manuscript Number**	58791
**(based on CONSORT-EHEALTH V1.6), available at [http://tinyurl.com/consort-ehealth-v1-6].**		
**Date completed**		
4/1/2024 3:45:09		
**by**		
Boyoung Kang		
Exploring the Effectiveness of Digital Interventions for Loneliness and Depression among College Students: A Mixed-Methods Study		
TITLE		
**1a-i) Identify the mode of delivery in the title**		
Although the mode of delivery (i.e., mobile app) is not explicitly mentioned in the title of our paper, the intervention was delivered through a mobile application. Participants were instructed to download and install the app on their Android or iOS devices. Table 1 in my paper.		
**1a-ii) Non-web-based components or important co-interventions in title**		
**1a-iii) Primary condition or target group in the title**		
Yes "undergraduate and graduate students aged 18-27 were initially recruited through the university's portal. Inclusion criteria required participants to have loneliness scores of 34-40 or higher on the UCLA Loneliness Scale and depression scores of 6 or higher on the PHQ-9, with some flexibility based on the number of applicants"		
ABSTRACT		
**1b-i) Key features/functionalities/components of the intervention and comparator in the METHODS section of the ABSTRACT**		
a control group using Bondee, a metaverse social network messenger app (n=10) in my abstract		
**1b-ii) Level of human involvement in the METHODS section of the ABSTRACT**		
**1b-iii) Open vs. closed, web-based (self-assessment) vs. face-to-face assessments in the METHODS section of the ABSTRACT**		
**1b-iv) RESULTS section in abstract must contain use data**		
**1b-v) CONCLUSIONS/DISCUSSION in abstract for negative trials**		
INTRODUCTION		
**2a-i) Problem and the type of system/solution**		
yes, "Participant Characteristics and Baseline Measures<br><br>Table 2 presents the demographic and clinical variables of the participants at baseline. The initial sample consisted of 63 participants, with 10 excluded due to loneliness scores below 34. Over half of the participants exhibited moderately high or high levels of loneliness, and approximately 42% reported PHQ-9 scores above 10, indicating a high probability of major depression.<br>"		
**2a-ii) Scientific background, rationale: What is known about the (type of) system**		
yes, "Recent systematic reviews and meta-analyses have demonstrated the effectiveness of digital mental health interventions in reducing symptoms of depression and anxiety and improving overall well-being [36,43]. Digital interventions, such as mobile apps and online therapies, have shown effectiveness comparable to traditional face-to-face therapy in treating depression [44,45]. Moreover, these digital solutions offer accessibility and can reduce psychological burdens associated with anonymity and stigma, making them particularly appealing to digital-native college students [46].<br><br>The use of chatbots in digital interventions has been shown to reduce the social threat associated with disclosing mental health issues, which is particularly important in societies with strong stigmas against mental health, such as South Korea [47]. Anonymity allows users to express and explore their thoughts and emotions more freely, enhancing treatment effectiveness [48,49]. Furthermore, user-friendly chatbot designs can significantly increase user satisfaction and reuse intention for counseling services, with user emotional expression intentions and chatbot familiarity mediating these effects [50].<br><br>"		
**Does your paper address CONSORT subitem 2b?**		
yes, "This study aims to test the following hypotheses, based on the existing literature and theoretical frameworks:<br><br>1.Intervention Effect: The group participating in the digital intervention program (Woebot or Happify) will experience a significant decrease in loneliness and depression compared to the control group (Bondee app users). This hypothesis is based on previous research demonstrating the effectiveness of digital interventions in reducing symptoms of loneliness and depression [8,42].<br>2.Perceived Benefits of Action: The group that responded positively to the question "At the beginning of the experiment (early March), did you expect the experiment to reduce loneliness and depression even slightly?" will experience a greater reduction in loneliness and depression after participating in the intervention program compared to the group that responded negatively. This hypothesis is derived from the Health Belief Model [58], which suggests that individuals who perceive greater benefits from an action are more likely to engage in that action and experience positive outcomes.<br><br>3.Perceived Barriers to Action: The group that responded positively to the question "Do you think digital applications can be helpful in alleviating loneliness and depression if they are effectively designed?" will experience a greater reduction in loneliness and depression after participating in the intervention program compared to the group that responded negatively. This hypothesis is also based on the Health Belief Model [58], which posits that individuals who perceive fewer barriers to an action are more likely to engage in that action and experience positive outcomes.<br><br>4.Help-seeking Behavior: The group that responded positively to the question "Do you think you can control emotions such as loneliness and depression only through your own efforts and will, without the help or intervention of others such as friends, family, organizations, professionals, or digital applications?" (indicating low help-seeking behavior) will experience a greater reduction in loneliness and depression after participating in the intervention program compared to the group that responded negatively (indicating high help-seeking behavior). This hypothesis is based on the notion that individuals with low help-seeking behavior may benefit more from the digital interventions, as they may have fewer alternative resources for managing their symptoms of loneliness and depression [59].<br><br>"		
METHODS		
**3a) CONSORT: Description of trial design (such as parallel, factorial) including allocation ratio**		
yes, "Experimental Design<br><br>This experimental study aims to measure the effectiveness of a digital intervention in reducing loneliness and depression indices in young adults experiencing loneliness and depression symptoms. The study also explores the perceived needs and areas for improvement through qualitative surveys and interviews on the content and satisfaction of the intervention. The research unfolds over a four-month period, spanning March to June 2023, with participants drawn from Sungkyunkwan University's Colleges of Natural Sciences and Humanities and Social Sciences in Seoul, South Korea.<br>"		
**3b) CONSORT: Important changes to methods after trial commencement (such as eligibility criteria), with reasons**		
We didn't change methods; but we included the graduate students up to age 27 since not sufficient number of students applied for our recruit.		
**3b-i) Bug fixes, Downtimes, Content Changes**		

**4a) CONSORT: Eligibility criteria for participants**		
Yes, A total of 63 undergraduate and graduate students aged 18-27 were initially recruited through the university's portal. Inclusion criteria required participants to have loneliness scores of 34-40 or higher on the UCLA Loneliness Scale and depression scores of 6 or higher on the PHQ-9, with some flexibility based on the number of applicants.		
**4a-i) Computer / Internet literacy**		
**4a-ii) Open vs. closed, web-based vs. face-to-face assessments:**		
yes, web-based assessment of PHQ9 and UCLA loneliness index		
**4a-iii) Information giving during recruitment**		
**4b) CONSORT: Settings and locations where the data were collected**		
yes, "Assessment Tools and Data Collection<br>Quantitative data were collected through pre- and post-intervention assessments using the UCLA Loneliness Scale and the Patient Health Questionnaire-9 (PHQ-9).<br>In this section, clarity in the description of assessment tools and data collection procedures is ensured. The UCLA Loneliness Scale and PHQ-9 were administered to quantify loneliness and depressive symptoms, respectively. Specifically, the UCLA Loneliness Scale 3rd revised edition Korean version was utilized to assess loneliness, while the Korean-translated version of the PHQ-9 was employed to measure depressive symptoms. These tools were selected based on their established validity and reliability in assessing mental health indicators in the Korean population.<br><br>"		
**4b-i) Report if outcomes were (self-)assessed through online questionnaires**		
Yes, Online assessed (PHQ9 and UCLA,) but self-report assessing(Mental health literacy), "Assessment Tools and Data Collection<br>Quantitative data were collected through pre- and post-intervention assessments using the UCLA Loneliness Scale and the Patient Health Questionnaire-9 (PHQ-9).<br>In this section, clarity in the description of assessment tools and data collection procedures is ensured. The UCLA Loneliness Scale and PHQ-9 were administered to quantify loneliness and depressive symptoms, respectively. Specifically, the UCLA Loneliness Scale 3rd revised edition Korean version was utilized to assess loneliness, while the Korean-translated version of the PHQ-9 was employed to measure depressive symptoms. These tools were selected based on their established validity and reliability in assessing mental health indicators in the Korean population.<br>"		
**4b-ii) Report how institutional affiliations are displayed**		
**5) CONSORT: Describe the interventions for each group with sufficient details to allow replication, including how and when they were actually administered**		
**5-i) Mention names, credential, affiliations of the developers, sponsors, and owners**		
**5-ii) Describe the history/development process**		
**5-iii) Revisions and updating**		
**5-iv) Quality assurance methods**		
**5-v) Ensure replicability by publishing the source code, and/or providing screenshots/screen-capture video, and/or providing flowcharts of the algorithms used**		
**5-vi) Digital preservation**		
**5-vii) Access**		
Only free version was available to students, see table 6 in my paper.		
**5-viii) Mode of delivery, features/functionalities/components of the intervention and comparator, and the theoretical framework**		
yes, "Intervention<br><br>Given the importance of selecting effective digital interventions for addressing loneliness and depression among college students, Woebot and Happify were chosen for their demonstrated efficacy in prior research. Woebot, a chatbot based on CBT principles, has shown effectiveness in reducing symptoms of depression and anxiety among young adults, as evidenced by studies such as the randomized controlled trial [42]. Additionally, Happify has demonstrated promise in alleviating loneliness, particularly during challenging periods such as the COVID-19 pandemic. Qualitative focus group discussions[39]. Moreover, both Woebot and Happify are readily accessible for download in Korea, providing convenient and accessible platforms for individuals seeking to improve their mental well-being. These chatbots were also selected based on the performance results of mental health chatbots that have been verified for effectiveness and satisfaction in clinical experimental studies." See Fig 1 also. "Intervention Effects and Trends<br><br>The quantitative data analysis results showed a slight decrease in loneliness and depression after the intervention, as shown in Figure 2. However, these effects did not reach statistical significance, and one of the main reasons for this is thought to be the small sample size. In particular, the number of Bondee app users in the control group decreased by more than half (baseline: n=10; post: n=3), making it statistically challenging to compare with the control group. Therefore, it can be said that the hypothesized intervention effect (Hypothesis 1) was not verified.<br>"		
**5-ix) Describe use parameters**		
**5-x) Clarify the level of human involvement**		
**5-xi) Report any prompts/reminders used**		
During the 3-month experiment, we sent encouragement messages at least once a month via KakaoTalk, a popular Korean messaging app, reminding participants to submit screenshots of their completed tasks. As mentioned in the paper, we continuously informed participants that they would receive incentive rewards if they remained in the experiment until the end." Participants were compensated with a final success participation incentive of 30 US dollars upon completion of the study, aiming to enhance retention and encourage continued engagement with the intervention.<br><br>"		
**5-xii) Describe any co-interventions (incl. training/support)**		
Although not included in this paper, the submitted protocol contains a user manual for the app.https://docs.google.com/document/d/1nNIu_WMEcJJWdURFzBZJ7LshOCsUdW1Rmmv-wgzJelg/edit?usp=sharing		
**6a) CONSORT: Completely defined pre-specified primary and secondary outcome measures, including how and when they were assessed**		
yes, "Inclusion criteria required participants to have loneliness scores of 34-40 or higher on the UCLA Loneliness Scale and depression scores of 6 or higher on the PHQ-9, with some flexibility based on the number of applicants. Participants with very low loneliness or depression scores were excluded, as well as those not confident in their English skills. "		
**6a-i) Online questionnaires: describe if they were validated for online use and apply CHERRIES items to describe how the questionnaires were designed/deployed**		
**6a-ii) Describe whether and how "use" (including intensity of use/dosage) was defined/measured/monitored**		
**6a-iii) Describe whether, how, and when qualitative feedback from participants was obtained**		
**6b) CONSORT: Any changes to trial outcomes after the trial commenced, with reasons**		

yes, "Assessment Tools and Data Collection
Quantitative data were collected through pre- and post-intervention assessments using the UCLA Loneliness Scale and the Patient Health Questionnaire-9 (PHQ-9).
In this section, clarity in the description of assessment tools and data collection procedures is ensured. The UCLA Loneliness Scale and PHQ-9 were administered to quantify loneliness and depressive symptoms, respectively. Specifically, the UCLA Loneliness Scale 3rd revised edition Korean version was utilized to assess loneliness, while the Korean-translated version of the PHQ-9 was employed to measure depressive symptoms. These tools were selected based on their established validity and reliability in assessing mental health indicators in the Korean population.

"

7a) CONSORT: How sample size was determined

7a-i) Describe whether and how expected attrition was taken into account when calculating the sample size

7b) CONSORT: When applicable, explanation of any interim analyses and stopping guidelines

yes, "Inclusion criteria required participants to have loneliness scores of 34-40 or higher on the UCLA Loneliness Scale and depression scores of 6 or higher on the PHQ-9, with some flexibility based on the number of applicants. Participants with very low loneliness or depression scores were excluded, as well as those not confident in their English skills. "

8a) CONSORT: Method used to generate the random allocation sequence

During the randomized trial, participants were allocated to ensure an equal distribution of gender, age, depression scores, and loneliness scores across the study groups.See fig1.

8b) CONSORT: Type of randomisation; details of any restriction (such as blocking and block size)

During the randomized trial, participants were allocated to ensure an equal distribution of gender, age, depression scores, and loneliness scores across the study groups.See fig1.

9) CONSORT: Mechanism used to implement the random allocation sequence (such as sequentially numbered containers), describing any steps taken to conceal the sequence until interventions were assigned

Figure 1 illustrates the flow of participants through the study. Of the initial 63 respondents, 10 were excluded due to loneliness scores below 34. The remaining 53 participants were randomly assigned to the intervention groups (Woebot: n=22; Happify: n=21) and the control group (Bondee: n=10). Due to attrition, the final sample consisted of 27 participants (Woebot: n=15; Happify: n=9; Bondee: n=3).

10) CONSORT: Who generated the random allocation sequence, who enrolled participants, and who assigned participants to interventions

Leading author, Boyoung Kang

11a) CONSORT: Blinding - If done, who was blinded after assignment to interventions (for example, participants, care providers, those assessing outcomes) and how

11a-i) Specify who was blinded, and who wasn't

In this study, participants were randomly assigned to either the Woebot or Happify groups for the digital therapy intervention. However, the control group was assigned to a non-digital therapy app (Bondee, a metaverse app) and was aware of this allocation. As a result, it was not possible to blind the participants to their group assignment.

Due to the nature of the intervention, which involved using different apps, it was not feasible to blind the participants. However, the researchers who conducted the data analysis were blinded to the group allocations to minimize potential bias in the analysis and interpretation of the results.

While the lack of participant blinding is a limitation of this study, it is a common challenge in web-based and app-based trials, where participants are typically aware of the intervention they are receiving. This limitation should be clearly acknowledged in the discussion section of the paper.

11a-ii) Discuss e.g., whether participants knew which intervention was the "intervention of interest" and which one was the "comparator"

11b) CONSORT: If relevant, description of the similarity of interventions

This item is not directly applicable to our study, as it typically refers to the similarity between a placebo or sham intervention and an active intervention in medication trials. However, we can describe the similarities and differences between the interventions used in our study.

Participants in the intervention groups were assigned to use either Woebot or Happify, which are both digital therapy apps designed to support mental well-being. While these apps share the common goal of improving mental health, they differ in their approach and features. Woebot is a chatbot-based app that uses cognitive-behavioral therapy (CBT) techniques, whereas Happify employs various evidence-based strategies, including CBT, mindfulness, and positive psychology.

The control group was assigned to use Bondee, a metaverse app not specifically designed for mental health support. This app serves as a non-therapeutic control condition, allowing for comparisons between the digital therapy interventions and a neutral app experience.

12a) CONSORT: Statistical methods used to compare groups for primary and secondary outcomes

"the researcher used Welch's ANOVA, a statistical test method that can be used when the sample sizes are unbalanced (Bondee: n=3; Woebot: n=15; Happify: n=9), to analyze the trends in the UCLA Loneliness Scale and PHQ-9 Depression Scale scores. Welch's ANOVA was chosen because it is robust to violations of the assumption of equal variances across groups, which is likely to occur with unequal sample sizes [54]. The following results were obtained for the UCLA Loneliness Scale and PHQ-9 Depression Scale scores through the analysis of scores." in my paper.

12a-i) Imputation techniques to deal with attrition / missing values

:the researcher used Welch's ANOVA, a statistical test method that can be used when the sample sizes are unbalanced (Bondee: n=3; Woebot: n=15; Happify: n=9), to analyze the trends in the UCLA Loneliness Scale and PHQ-9 Depression Scale scores. Welch's ANOVA was chosen because it is robust to violations of the assumption of equal variances across groups, which is likely to occur with unequal sample sizes [54]. The following results were obtained for the UCLA Loneliness Scale and PHQ-9 Depression Scale scores through the analysis of scores." in my paper. "

12b) CONSORT: Methods for additional analyses, such as subgroup analyses and adjusted analyses

Hypotheses testing, "However, the Mann-Whitney U test, used due to the small sample size and non-normal distribution of the data (Nachar, 2008), did not reveal statistically significant differences between the two groups (UCLA Loneliness: U=114.5, p=0.4616; PHQ-9: U=90.0, p=0.7286). Despite the non-significant results, there is a subtle trend in UCLA loneliness scores related to perceived benefits of action.

"

RESULTS

13a) CONSORT: For each group, the numbers of participants who were randomly assigned, received intended treatment, and were analysed for the primary outcome

UCLA Loneliness Scale:<br><br>Bondee: A slight decrease in score, indicating a positive effect.<br>Woebot: The score decreased initially and then increased slightly during the intervention period.<br>Happify: The score decreased both during and after the intervention period, indicating a positive effect.<br><br>PHQ-9 Depression Scale:<br><br>Bondee: A slight decrease in score, indicating a positive effect.<br>Woebot: The score decreased initially and then increased slightly during the intervention period.<br>Happify: The score decreased both during and after the intervention period, indicating a positive effect.		
**13b) CONSORT: For each group, losses and exclusions after randomisation, together with reasons**		
For each group (Woebot, Happify, and control), we report the number of participants lost to follow-up or excluded after randomization, along with the reasons for these losses or exclusions. see fig 1 and discussion, "Additionally, the demanding academic schedule and competitive environment experienced by university students, coupled with the ongoing stressors of daily life, could have exacerbated feelings of depression and loneliness, mitigating the effectiveness of the intervention in reducing these indices. It's worth noting that the experiment period, especially from March to June in Korea, coincides with the beginning of the academic year for college students. This transition period may have introduced additional stressors as students adapt to new academic challenges and social environments, potentially influencing their mental health during the intervention period. These contextual factors may have influenced the observed trends in loneliness and depression scores, highlighting the complex interplay between intervention efficacy and participants' socio-environmental circumstances. "		
**13b-i) Attrition diagram**		
**14a) CONSORT: Dates defining the periods of recruitment and follow-up**		
"This experimental study aims to measure the effectiveness of a digital intervention in reducing loneliness and depression indices in young adults experiencing loneliness and depression symptoms. The study also explores the perceived needs and areas for improvement through qualitative surveys and interviews on the content and satisfaction of the intervention. The research unfolds over a four-month period, spanning March to June 2023, with participants drawn from Sungkyunkwan University's Colleges of Natural Sciences and Humanities and Social Sciences in Seoul, South Korea."		
**14a-i) Indicate if critical "secular events" fell into the study period**		
**14b) CONSORT: Why the trial ended or was stopped (early)**		
2months intervention and one month post intervention. "<br>This experimental study aims to measure the effectiveness of a digital intervention in reducing loneliness and depression indices in young adults experiencing loneliness and depression symptoms. The study also explores the perceived needs and areas for improvement through qualitative surveys and interviews on the content and satisfaction of the intervention. The research unfolds over a four-month period, spanning March to June 2023, with participants drawn from Sungkyunkwan University's Colleges of Natural Sciences and Humanities and Social Sciences in Seoul, South Korea.<br>"		
**15) CONSORT: A table showing baseline demographic and clinical characteristics for each group**		
See Table 2: Demographic and clinical variables of participants at baseline		
**15-i) Report demographics associated with digital divide issues**		
does not apply to my research since the participants are all same university students from age 18-27.		
**16a) CONSORT: For each group, number of participants (denominator) included in each analysis and whether the analysis was by original assigned groups**		
**16-i) Report multiple "denominators" and provide definitions**		
We report the following "denominators" and their corresponding sample sizes (N) for each group: see fig1 and table2.		
**16-ii) Primary analysis should be intent-to-treat**		
**17a) CONSORT: For each primary and secondary outcome, results for each group, and the estimated effect size and its precision (such as 95% confidence interval)**		
Hypothesis Testing<br><br>As hypothesized in the Method section, we investigated whether participants' initial beliefs regarding the potential effectiveness of the intervention influenced their outcomes.<br><br>Hypothesis 2: Belief in Benefits of Action<br>The graph (Figure 3) shows that the positive group, which answered positively to the question "Did you expect the experiment to reduce loneliness and depression even slightly at the beginning of the experiment (early March)?", had a higher reduction rate in loneliness and depression scores compared to the negative group. However, the Mann-Whitney U test, used due to the small sample size and non-normal distribution of the data (Nachar, 2008), did not reveal statistically significant differences between the two groups (UCLA Loneliness: U=114.5, p=0.4616; PHQ-9: U=90.0, p=0.7286). Despite the non-significant results, there is a subtle trend in UCLA loneliness scores related to perceived benefits of action.<br><br>Hypothesis 3: Belief in Barriers to Action<br>The results comparing the positive and negative groups for the question "Do you think digital applications can be helpful in alleviating loneliness and depression if they are effectively designed?" showed a slightly larger difference for PHQ-9 than for the UCLA index, but these differences were not statistically significant (UCLA Loneliness: U=95.5, p=0.9258; PHQ-9: U=113.5, p=0.4759). Although the p-values did not reach significance, there is a trend in PHQ-9 scores related to barrier perception.<br><br>Hypothesis 4: Self-Help Behavior<br>The results for the positive and negative groups for the question "Do you think you can control emotions such as loneliness and depression only through your own efforts and will, without the help or intervention of others such as friends, family, organizations, professionals, or digital applications?" are as follows:<br><br>UCLA Loneliness:<br>- Mann-Whitney U test result: U=112.0, p-value=0.7624<br>- The non-significant p-value suggests no clear difference in UCLA loneliness scores related to attitude towards help-seeking behavior.<br>PHQ-9:<br>- Mann-Whitney U test result: U=121.5, p-value=0.4723<br>- Although the p-value did not reach statistical significance, there is a subtle trend in PHQ-9 scores related to attitude towards help-seeking behavior		
**17a-i) Presentation of process outcomes such as metrics of use and intensity of use**		
**17b) CONSORT: For binary outcomes, presentation of both absolute and relative effect sizes is recommended**		
Due to the small sample sizes in our study (Woebot: 15, Happify: 9, Control: 3), we did not present both absolute and relative effect sizes for binary outcomes. The small number of participants in each group, particularly in the control group, would lead to imprecise estimates of effect sizes and wide confidence intervals, which could be misleading.<br><br>Instead, we focused on reporting the observed frequencies and percentages for binary outcomes in each group. While this approach does not provide a direct comparison of effect sizes between groups, it offers a transparent representation of the data given the limitations imposed by the small sample sizes.		
**18) CONSORT: Results of any other analyses performed, including subgroup analyses and adjusted analyses, distinguishing pre-specified from exploratory**		

In addition to the quantitative analyses reported for the primary and secondary outcomes, we conducted a qualitative analysis to explore participants' experiences and perceptions of the digital interventions (Woebot and Happify) and the control condition (Bondee).

Interpretation of Non-Significant Results
It's crucial to delve into the implications and insights gained from non-significant findings. Despite the small sample size leading to non-significance in the quantitative results, qualitative insights from the survey provided valuable information regarding participants' experiences with the apps, including practical benefits and potential implications for future interventions. Additionally, the qualitative analysis will shed light on the reasons behind 'somewhat satisfied' and 'not satisfied' ratings, offering further context and understanding.

Qualitative Findings
The qualitative findings from the open-ended survey questions and focus group interviews provided valuable insights into participants' experiences with the digital interventions and their coping mechanisms for loneliness.

18-i) Subgroup analysis of comparing only users

19) CONSORT: All important harms or unintended effects in each group

In the informed consent document, we clearly stated the potential risks and unintended effects associated with the intervention experiments and focus group interviews. We also outlined the measures we would take to guide participants to appropriate resources if they experienced any psychiatric symptoms during the study.

Throughout the study, we monitored participants for any signs of adverse events or unintended effects. No serious harms or unintended effects were reported in any of the study groups (Woebot, Happify, or control).

19-i) Include privacy breaches, technical problems

19-ii) Include qualitative feedback from participants or observations from staff/researchers

DISCUSSION

20) CONSORT: Trial limitations, addressing sources of potential bias, imprecision, multiplicity of analyses

20-i) Typical limitations in ehealth trials

This study has several limitations that are common in eHealth trials.

First, participants were not blinded to the intervention they received, as is often the case in eHealth studies. This lack of blinding could have introduced bias, as participants' expectations and beliefs about the interventions may have influenced their responses and outcomes.

Second, we looked at multiple outcomes, including depression, loneliness, and several process outcomes (e.g., engagement, satisfaction). This multiplicity of outcomes increases the risk of a Type I error, where a statistically significant result may be found by chance. To mitigate this risk, we focused our interpretation on the primary outcomes and considered the secondary and exploratory outcomes as hypothesis-generating rather than confirmatory.

Third, the study may have been affected by biases related to non-use of the interventions or usability issues. Some participants, particularly in the control group, reported low levels of engagement with their assigned app. This lack of engagement could have undermined the potential effects of the interventions and introduced bias in the comparison between groups. Future studies should aim to optimize the design and delivery of digital interventions to maximize engagement and adherence.

Fourth, the informed consent procedures may have introduced bias by influencing participants' expectations and behaviors. Participants were aware that they were participating in a study of digital mental health interventions, which could have led to a heightened awareness of their mental health symptoms and a greater motivation to engage with the interventions. This potential bias should be considered when interpreting the results.

Finally, unexpected events, such as technical difficulties with the apps or changes in participants' personal circumstances, could have affected the study outcomes. Although we did not encounter any major technical issues during the study, it is possible that some participants may have experienced difficulties that were not reported to the research team.

In conclusion, while this study provides valuable insights into the potential of digital mental health interventions, the findings should be interpreted in light of these limitations. Future research should aim to address these limitations by using more robust study designs, such as blinded randomized controlled trials, and by implementing strategies to minimize biases related to non-use, usability, and informed consent procedures.

21) CONSORT: Generalisability (external validity, applicability) of the trial findings

21-i) Generalizability to other populations

21-ii) Discuss if there were elements in the RCT that would be different in a routine application setting

22) CONSORT: Interpretation consistent with results, balancing benefits and harms, and considering other relevant evidence

22-i) Restate study questions and summarize the answers suggested by the data, starting with primary outcomes and process outcomes (use)

The primary aim of this study was to investigate the effectiveness of two digital mental health interventions (Woebot and Happify) compared to a control condition (Bondee) in reducing depression and loneliness among university students. The secondary aim was to explore participants' experiences and perceptions of these interventions.

Regarding the primary outcomes, our findings suggest that both Woebot and Happify may be more effective than the control condition in reducing symptoms of depression and loneliness. However, due to the small sample size and high attrition rates, these results should be interpreted with caution. Further research with larger sample sizes and more robust designs is needed to confirm the effectiveness of these interventions.

In terms of process outcomes (use), participants in the Woebot and Happify groups reported higher levels of engagement and satisfaction compared to those in the control group. This suggests that the digital mental health interventions were more acceptable and feasible than the non-therapeutic control app.

The qualitative findings provide additional insights into participants' experiences with the interventions. Participants in the Woebot and Happify groups reported that the apps helped them to develop coping strategies, gain self-awareness, and feel supported. However, some participants also expressed concerns about the limitations of digital interventions, such as the lack of human connection and the potential for over-reliance on the apps.

Overall, our findings provide preliminary evidence for the potential of digital mental health interventions in reducing depression and loneliness among university students. However, the limitations of the study, including the small sample size, high attrition rates, and lack of long-term follow-up, should be addressed in future research.

Further studies are needed to investigate the effectiveness of these interventions in larger and more diverse samples, to explore the mechanisms of change underlying their effects, and to assess their long-term impact on mental health outcomes. Additionally, future research should aim to identify the factors that influence engagement with and adherence to digital mental health interventions, in order to optimize their design and delivery.

22-ii) Highlight unanswered new questions, suggest future research

Other information

23) CONSORT: Registration number and name of trial registry

Clinical Research Information Service (CRIS, https://cris.nih.go.kr/)

24) CONSORT: Where the full trial protocol can be accessed, if available

We're in the process of retrospectively registering the trial with the Clinical Research Information Service (CRIS, https://cris.nih.go.kr/) in South Korea.. Meanwhile you can access to our protocol here: https://docs.google.com/document/d/1nNlu_WMEcJJWdURFzBZJ7LshOCsUdW1Rmmv-wgzJelg/edit?usp=sharing

25) CONSORT: Sources of funding and other support (such as supply of drugs), role of funders

This research was conducted with funding from the K-medi global talent development project of the Ministry of Health and Welfare of the Republic of Korea. Assignment number: HI22C2185

X26-i) Comment on ethics committee approval

x26-ii) Outline informed consent procedures

X26-iii) Safety and security procedures

X27-i) State the relation of the study team towards the system being evaluated
