# Supplementary figures and images for: Digital Interventions for Reducing Loneliness and Depression in Korean College Students: Mixed Methods Evaluation
Source: JMIR Form Res. 2024 Sep 12;8:e58791. doi: 10.2196/58791 (PMC11427852; doi:10.2196/58791)

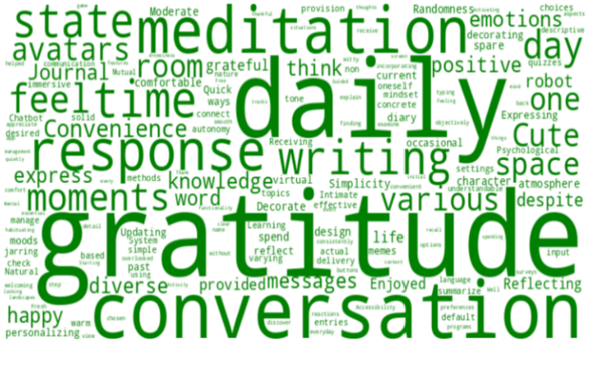

Supplement: Multimedia Appendix 9 [file formative_v8i1e58791_app9.png]
